# ﻿Taxonomic revision of the *Erigeronacris* group (Asteraceae) in Murmansk Region, Russia, reveals a complex pattern of native and alien taxa

**DOI:** 10.3897/phytokeys.235.111020

**Published:** 2023-11-15

**Authors:** Alexander N. Sennikov, Mikhail N. Kozhin

**Affiliations:** 1 Botanical Museum, Finnish Museum of Natural History, University of Helsinki, Helsinki 00014, Finland University of Helsinki Helsinki Finland; 2 Avrorin Polar-Alpine Botanical Garden-Institute of Kola Science Centre of the Russian Academy of Sciences, Apatity 184209, Russia Avrorin Polar-Alpine Botanical Garden-Institute of Kola Science Centre of the Russian Academy of Sciences Apatity Russia

**Keywords:** Compositae, Kola Peninsula, Lapland, mapping, nomenclature, plant invasions, Pomors, taxonomy

## Abstract

Based on the evidence of morphology and a comprehensive revision of herbarium collections and field records, the taxonomy of the *Erigeronacris* group in Murmansk Region, European Russia, is completely revised. Its accepted diversity is increased from 2 to 8 taxa, including putative hybrids. The only native species, *E.politus*, is distributed in mountainous regions, along sea coasts and in the Kutsa River basin. Five species are alien: *E.rigidus* (previously confused with *E.acris* s.str.), *E.acris* s.str. (first recorded in the narrow taxonomic definition), *E.brachycephalus* (previously unrecorded), *E.droebachiensis* and *E.uralensis* (previously reported in error). Two major waves of the introduction of alien taxa are discovered, with different occurrences and species compositions. Regional and local dispersal by pomors (historical Russian settlers) occurred during their colonisation and traditional activities since the 12^th^ century (archaeophytes or early neophytes); such alien taxa (*E.rigidus*, *E.brachycephalus*, and partly *E.acris*) are particularly common within the territory traditionally settled by Russian colonists but also found elsewhere along historical trade routes. Other alien species of the *E.acris* group (*E.droebachiensis*, *E.uralensis*, and partly *E.acris* and *E.brachycephalus*) colonised industrial areas in the 1960s–1990s as seed contaminants introduced during revegetation of slag dumps, stockyards, dams and channels. Putative hybrids between *E.politus* (native), *E.rigidus* and *E.acris* (aliens) are found in the places of co-occurrence. Updated nomenclature, synonymy and descriptions are provided for all accepted taxa.

## ﻿Introduction

Although a modern comprehensive inventory of the flora of Murmansk Region (European Russia) is still lacking, its vascular plants are relatively well known due to the 200-years-long history of botanical studies in this territory (Kozhin et al. 2020a). However, many taxonomically critical taxa still require revision in this territory, in order to elucidate their diversity and distributions. Besides, alien plants of Murmansk Region have never been at the focus of a dedicated study (Kozhin and Sennikov 2022), and require not only a special effort for their data mobilisation from the vast corpus of grey literature but also reassessment of their residence status (native vs. alien) because many archaeophytes or other long-term residents have been traditionally considered within the native component of the flora (Kozhin et al. in prep.).

One small, yet taxonomically unresolved group is a complex of *Erigeronacris* L. s.l. It belongs to E.sect.Trimorpha (Cass.) DC. This section is distinct due to the presence of three types of flowers in the capitula: tubular flowers in the centre, ray flowers without a lamina in the middle, and ray flowers with a short lamina at the margin (Nesom 2008). Among European taxa of this section, *E.acris* s.l. differs by its very short ligules and monocarpic life form (Halliday 1976).

The taxonomic diversity in the *E.acris* group seems to be maintained by self-pollination (Noyes 2000); the resulting taxa are stable in largely sympatric areas (Olander and Tyler 2017) and may therefore be treated at the rank of species (e.g. Tzvelev 1994). However, since diagnostic characters are very meagre in this taxonomic group, whose diversity has probably resulted from extensive hybridization (Tzvelev 1994; Olander and Tyler 2017), the rank of subspecies was also employed (e.g. Kurtto and Väre 1998; Olander and Tyler 2017).

The taxonomy of *E.acris* L. s.l. in Murmansk Region and neighbouring territories has been controversially treated. Orlova (1966) accepted two taxa, i.e. *E.acris* with hairy phyllaries, leaves and stems, and the nearly glabrous *E.politus* Fr. Kurtto and Väre (1998) recognized only one taxon in Finnish Lapland, E.acrissubsp.politus (Fr.) H.Lindb., a less hairy plant with rather few capitula and pinkish phyllaries. Tzvelev (1990, 1994) revised the taxonomy of *E.acris* s.l. in Eastern Europe; he accepted three taxa in Murmansk Region: *E.acris* s.str., whose synflorescence branches are densely covered by long simple hairs, *E.uralensis* Less. (syn. *E.brachycephalus* H.Lindb.), whose synflorescence branches are subglabrous or covered by short simple hairs, and *E.politus* Fr. (syn. *E.elongatus* Ledeb.), which embraces lower-sized subglabrous plants with longer branches and fewer capitula. Tzvelev also suggested that the name *E.decoloratus* may belong to populations intermediate between *E.politus* and *E.uralensis*, which do not deserve taxonomic separation from the latter. Olander and Tyler (2017) revised this group in Sweden and accepted three taxa, of which E.acrissubsp.acris is ubiquitous but more abundant in the south, with involucral bracts almost completely covered by simple hairs, E.acrissubsp.droebachiensis (O.F.Müll.) Mela (central Sweden) with pale phyllaries up to 5 mm long, numerous capitula and inconspicuous ray flowers, and E.acrissubsp.politus (northern Sweden) with darker phyllaries over 5 mm long, fewer capitula and well exserted ray flowers. They also indicated that the name E.acrissubsp.decoloratus (H.Lindb.) Hiitonen may belong to hybrids between E.acrissubsp.acris and the other two subspecies.

Hybrids in the *E.acris* group have long been reported or suspected, including those between hairy and glabrous taxa (e.g. Blytt 1906; Thellung 1923; Šída 1998, 2000, 2004), although some of these tentative reports (Botschantzev 1959; Tzvelev 1994) appeared to have mistaken unrecognised or synonymised taxa for hybrids.

Taxonomic opinions about the subdivision of *E.acris* s.l. differed widely to the extent that the treatments covering the same territory or closely neighbouring areas may be largely incongruent in the number of accepted taxa and their delimitation and diagnostic characters. This discrepancy urged us to revise the taxonomy of this group in Murmansk Region, in order to bridge together the existing treatments and to uncover the diversity and distribution patterns of the taxa involved. We also wanted to evaluate the resident status of these taxa in Murmansk Region because of their strong association with human dispersal (Kurtto and Väre 1998).

The present work provides a detailed treatment of the *E.acris* group for Murmansk Region but includes the whole history of its studies and involves comparisons with all the relevant taxa recognised in Fennoscandia, and places this study in the European context. It is considered a step towards a new revision of this difficult taxonomic group in Eastern Fennoscandia, which is a long and complicated process.

## ﻿Materials and methods

### ﻿Study area

**Figure 1.**
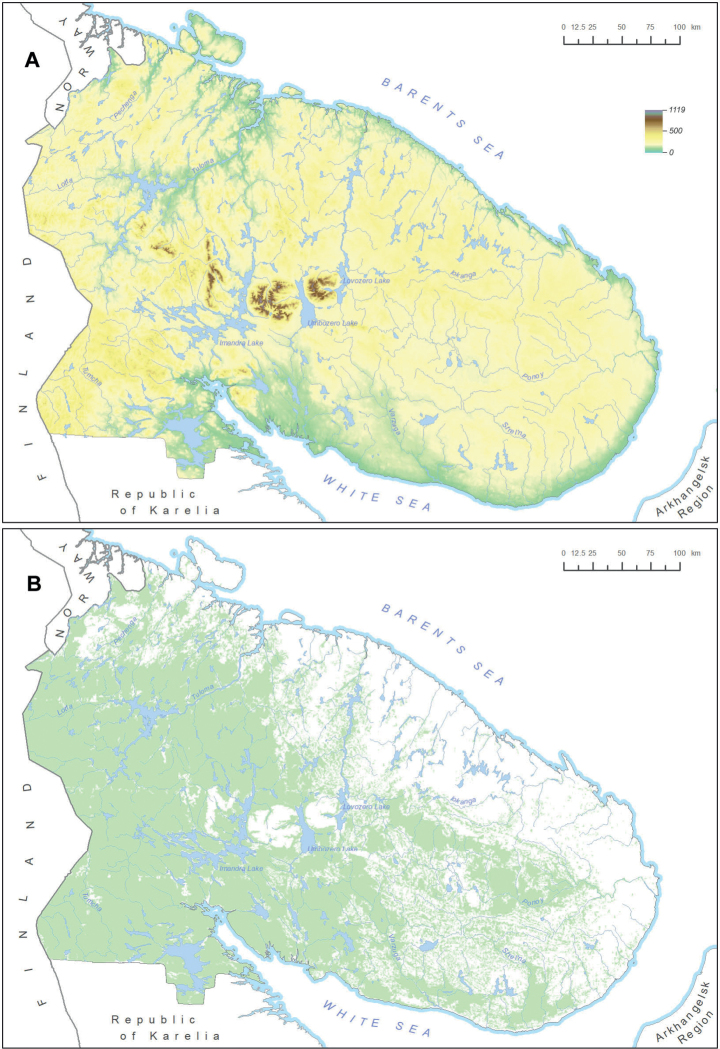
Study area: Murmansk Region, Russia **A** hypsometric map, major rivers and lakes **B** distribution of forested landscapes (denoted by green colour). Maps were created using ArcGIS software by Esri. ArcGIS is the intellectual property of Esri and is used herein under license. Copyright Esri. All rights reserved.

Murmansk Region is a top-level federal subject of the Russian Federation, situated in the north-western part of European Russia; this territory is also known as Russian Lapland in historical literature. It is largely situated on the Kola Peninsula, surrounded by the Barents Sea in the north and by the White Sea in the south. Its total area constitutes 144,902 km^2^. This territory is part of the Subarctic Zone; its vegetation is represented by tundra in the north, forest tundra in the major part of the mainland, and northern taiga in the south-west, next to the borders with Finland and Russian Karelia (Chernov 1971). The relief is largely flat and nearly monotonous, except for rocky mountain groups in the western part of the territory, among which the Khibiny and Lovozero Mts. are the highest to reach the maximum of 1191 m above sea level (Fig. 1A). Smaller but important hills and isolated outcrops are situated in the Kovdor and Kandalaksha Districts, whereas the north-western coast is incised by fjords and the other coastal areas are traversed by deeper river valleys. Forested areas are extensive in the western part, and forests are significantly present in the basins of the Ponoi River and the rivers flowing into the southern coastal waters of the White Sea (Fig. 1B).

### ﻿Material examined

This study was based on a comprehensive sampling of all herbarium specimens available from the study area and kept at H, INEP, KAND, KPABG, LE, LECB, MW, OULU, PTZ, TROM (herbarium acronyms according to Index Herbariorum (Thiers 2023)) and the Herbarium of the Apatity Branch of the Murmansk Arctic University (unregistered, provisional acronym ARCT). Documented observations (iNaturalist 2023) were also used. The specimens (258) and observations (3) were georeferenced and databased, and their data were made available as a taxonomic dataset (Sennikov and Kozhin 2023), which includes 261 herbarium specimens and documented observations altogether. Point distribution maps were generated in ArcGis 10.3.1 (https://www.esri.com) from the database, taking into account the residence status and period of introduction of the species in every locality; we distinguished three categories of species residence: native, pre-industrial alien (introduced prior to the industrialisation in the USSR, i.e. before the 1930s) and industrial alien (introduced with or after the industrialisation in the USSR, sometimes after 1930 but usually after 1960).

The herbarium specimens were examined taxonomically by A. Sennikov either *de visu* (H, OULU), or as high-quality scanned images (ARCT, INEP, KAND, KPABG, LE, MW), or as low-quality scanned images (TROM), or as photographs (LECB, PTZ). Scanned images were also used as illustrations.

### ﻿Nomenclature and bibliography

Special effort was used to trace validly published names at the level of species and subspecies, in order to produce a stable synonymy whenever the species or subspecies rank is preferred. More precise publication dates were traced from a variety of bibliographic sources, which helped to establish the sequence of publication and to ensure priority in difficult cases.

### ﻿Diagnostic characters

As in the previous revisions (Tzvelev 1994; Kurtto and Väre 1998; Šída 1998, 2000), we examined the following diagnostic characters: stem and phyllary colouration; stem, leaf and phyllary pubescence; length and density of pubescence; number, shape and density of cauline leaves; shape of synflorescence; ligulate flower colour. The value of individual characters may be limited due to high genetic variability or phenotypic plasticity, whereas a complex of characters may unambiguously characterise the taxa.

In this work, we used a classical method of morphological comparisons, observing discontinuities in plant variability. We did not use statistical methods (like employed by Olander and Tyler (2017)) because, in order to distinguish between very closely related taxa, infra- and interpopulational variability must be considered separately, whereas our material was represented by previously collected herbarium specimens, i.e. a random selection from multiple populations that do not allow to assess their interpopulational variability.

### ﻿Taxonomic concept and ranking

We follow the concept of narrowly defined taxa in the *Erigeronacris* group, which is universally adopted nowadays (Halliday 1976; Tzvelev 1994; Kurtto and Väre 1998; Šída 1998, 2000, 2004; Greuter 2006; Olander and Tyler 2017; PoWO 2023). This concept is justified because of the limited variability and morphological distinction of the accepted taxa. Ranking in this group is disputable; species level is currently accepted in the Czech Republic (Šída 1998, 2000, 2004) and Eastern Europe (Tzvelev 1994), whereas subspecies level is preferred in Finland (Kurtto and Väre 1998) and Sweden (Olander and Tyler 2017), and in European (Halliday 1976; Greuter 2006) and global (PoWO 2023) compilations. We accept species rank because the ranked taxa are clearly defined by morphology and commonly co-occur in the same territory with limited hybridisation, thus complying with the biological species concept due to apparent reproductive isolation (Gao and Rieseberg 2020).

### ﻿Classification of alien occurrences

In agreement with Pyšek et al. (2002), origin status and invasion status were determined for each accepted species, and residence status was determined for each recorded locality. We determined the origin status as native or alien; a taxon was treated as alien if it arrived to the territory with human assistance at any time period, including the remote past from which no historical plant records or other direct evidence are available. Each non-native species was classified according to its invasion status either as casual or naturalised, with further estimation of invasiveness (Richardson et al. 2000). Records of alien plants were classified according to their period of introduction (residence status), using the major subdivision between archaeophytes and neophytes as in Pyšek et al. (2002) and major periods of the recent political history as in Sennikov and Lazkov (2021). For temporal classification of neophyte alien records of the *Erigeronacris* group in Murmansk Region, we used the 1930s as a temporal limit; this limit reflects a major change in economic activities, transportation and human migrations, which was linked with the beginning of industrialisation in the USSR (Lewis 1979).

The history of introduction was determined based on the history of human activities in a certain locality and in the territory as a whole. The local history was obtained from historical accounts in cases of the distant past, or from technical reports and local knowledge in cases of the recent past. Local plant introductions were linked to the local human activities and their time periods. We cross-checked our information against the knowledge available from the neighbouring territories, i.e. Finland and Russian Karelia.

We inferred pathways of introduction for alien taxa, based on direct evidence as recorded by field collectors or on indirect evidence as derived from the local history. The pathways were categorised according to Hulme et al. (2008) and interpreted as recommended by Harrower et al. (2018).

## ﻿Results

### ﻿Overview of historical herbarium collections

The examined collections are comprehensive historical materials and include all periods of the botanical history in the present-day territory of Murmansk Region. Many of these collections were taken into account in various botanical publications. So far, no proper overview of botanical collections and their corresponding publications exist for Murmansk Region; for this reason, we provide a more detailed description of the *Erigeron* collections in order to uncover their link with historical publications and major events of the botanical exploration.

The first record of *Erigeronacris* s.l. from the Kola Peninsula was published by Jacob Fellman (1831), who reported this group from the south-western part of the territory. Nowadays, Jacob Fellman’s herbarium collection is fragmentary; its *reliquiae* are preserved at the University of Helsinki (Väre 2011). However, no specimens of *E.acris* s.l. survived in this collection.

The earliest historical specimens are available from Russian academic expeditions and the Finnish botanical exploration of the Kola Peninsula. The first extant specimens were collected by A.F. Middendorf in 1840 during his academic expedition along the Barents Sea coast (Sukhova and Tammiksaar 2015). Further collections were exclusively Finnish, linked with botanical explorations of Russian Lapland (Uotila 2013; Väre 2017). During 1861 and 1863, N.I. Fellman and his team made very important collections along the southern and eastern sea coasts and in Kola Town (Sennikov and Kozhin 2018), thus bringing evidence for an early introduction of alien plants by the Pomors. A.J. Malmberg in 1870 (Malmberg 1926; Lappalainen 1959), R.B. Enwald and C.A. Knabe in 1880 (Uotila 2013) acted as commercial botanical collectors and brought many well-documented specimens from the coastal areas. At the same time, geologist A. Göbel, who was dispatched by the Russian Academy of Sciences to Russian Lapland in 1868–1870, made some collections along the northern and eastern coasts but his botanical collections were very poorly prepared and extremely inaccurately documented. V.F. Brotherus (1886) collected in the western parts of the territory in 1885, and made the first good collections from the Rybachii Peninsula. These early explorations, which aimed at the primary floristic knowledge and focused largely on coastal areas with an emphasis on its western (Kandalaksha) and eastern (Ponoi) extremities, culminated with the Great Kola Expedition in 1887, 1889 and 1892, which was organised by the University of Helsinki and the Societas pro Fauna et Flora Fennica with the major aim to cover the interior parts of the Kola Peninsula (Rikkinen 1980; Uotila 2013). With these expeditions, the basic knowledge about native and archaeophytic populations of *E.acris* s.l. was obtained.

Further botanical expeditions focused on the “white spot” areas from which no botanical knowledge had been available. V. Borg and A. Rantaniemi extensively collected in Kuusamo, covering the territory of the Kutsa River and neighbouring villages (Uotila 2013). This important territory became a nature reserve and had been subsequently visited several times by various Finnish researchers before it was ceded to the USSR after the Second World War. More recently, it was revisited by T. Ulvinen who published a synopsis of the flora of the Kutsa Nature Reserve and its vicinities (Ulvinen 1996).

K. Regel made extensive explorations of plant communities in the Kola Peninsula but collected rather few specimens. We traced only two specimens of *E.acris* s.l. which he collected along the Ponoi River (Regel 1914, 1927). Another great early expedition focused on plant communities was made in 1927 by G. Zinserling, who collected many important specimens along the southern coast of the Kola Peninsula (Zinserling 1935).

The Polar-Alpine Botanical Garden-Institute was established in 1931 in the Khibiny Mts. This botanical institution triggered a new period of regular botanical studies of Murmansk Region. Eventually, these academic activities led to the five-volume book “Flora of Murmansk Region” (Gorodkov 1953; Poyarkova 1954, 1956, 1959, 1966), which was considered among the best regional synopses in the USSR. Distribution maps based on point occurrence data, which were provided for each species treated in this book, were digitised and made available online, including two accepted species of *E.acris* s.l. (Kozhin et al. 2020a).

Higher mountains of the western part of the Kola Peninsula were in focus of botanical studies in the 1930s, when their exploration for mining of natural resources had been initiated. This study was summarised by B. Mishkin in his monograph on the flora of the Khibiny Mts. (Mishkin 1953). The neighbouring Lovozero Mts. were studied in detail much later, during the 1970s–1980s (Belkina et al. 1991).

The Kandalaksha Bay, with its many islands, has been thoroughly explored for 75 years due to the existence of the Kandalaksha Nature Reserve (Kozhin and Sennikov 2020). The herbarium collections from its territory are kept also in a dedicated herbarium repository in the reserve.

Another nature reserve with a long-standing record of botanical explorations is Pasvik, situated at the border with Norway. Despite its tiny territory, its vascular plants were completely inventoried three times, but only the latest revision included records of *E.acris* s.l. (Kravchenko 2020). Alien plants of this territory were studied in the course of a transborder project that involved botanists from Norway, Finland and Russia (Alm et al. 1997).

As native vascular plants of Murmansk Region were considered rather sufficiently studied, their alien counterparts remained largely neglected (Kozhin et al. in prep.). During the latest 20 years, the effect of the revegetation of slag dumps in electric power stations and stockyards in mining factories was examined (Evdokimova et al. 2005; Kapelkina 2014; Timofeeva et al. 2016), however, without paying a proper attention to the introduction of alien plants. Their inventory in Murmansk Region has been started recently, with a few minor contributions published to date (Kozhin et al. 2020b; Kozhin and Sennikov 2022).

## ﻿Taxonomic synopsis

### ﻿1. Erigeron politus

Taxon classificationPlantaeAsteralesAsteraceae

Fr. in Bot. Not. 1843: 120 (1843)

58447CCD-4F87-5761-A678-09744964F7DC

Fig. 3

– Erigeronacrisvar.politus (Fr.) Mela, Lyhyk. Kasvioppi Kasvio, ed. 1: 66 (1877) – Erigeronacrissubvar.politus (Fr.) Mela, Lyhyk. Kasvioppi Kasvio, ed. 2: 79 (1884) – Erigerondroebachiensisvar.politus (Fr.) Mela, Suomen Kasvio, ed. 3: 174 (1895) – Erigeronacrissubsp.politus (Fr.) H.Lindb., Enum. Pl. Fennoscand. Orient.: 56 (1901).
= Erigeronelongatus Ledeb., Icon. Pl. Fl. Ross. 1: 9 (1829), nom. illeg., non Moench (1802) – Erigeronacrisvar.elongatus Herder in Bull. Soc. Imp. Naturalistes Moscou 38(2): 391 (1865) – Erigeronacrissubsp.elongatus (Herder) Kindb., Svensk Fl.: 296 (1877) – Erigeronacrisf.elongatus (Herder) Mela, Lyhyk. Kasvioppi Kasvio, ed. 1: 66 (1877) – Erigerondroebachiensissubsp.elongatus (Herder) Mela, Suomen Kasvio, ed. 3: 174 (1895). Type. Russia. “Altai”, 1826, Herb. Ledebour 1308 (lectotype LE 1043841, designated here; isolectotype LE 1043843).

#### Type.

**Figure 2.**
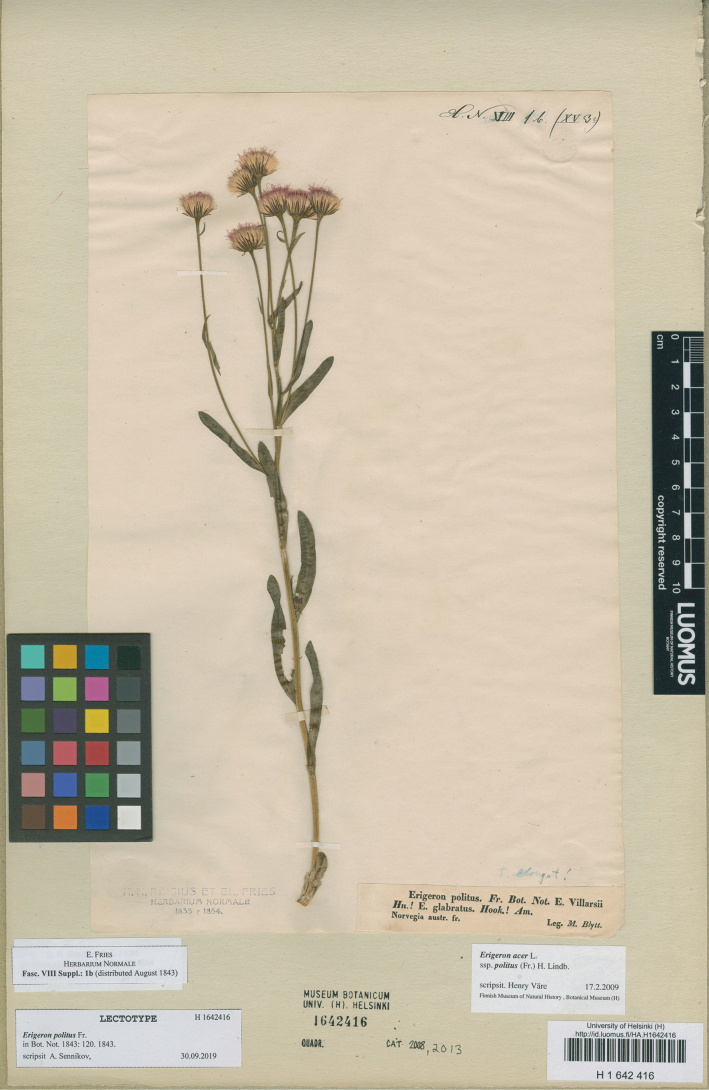
Lectotype of *Erigeronpolitus* Fr. (H 1642416). Courtesy of the Finnish Museum of Natural History, University of Helsinki.

Norway. “Norvegia austr. fr.,” *M. Blytt* [E.Fries, *Herbarium Normale* VIII: Suppl. no. 1b] (lectotype H 1642416, designated here). Fig. 2. Superseded neotype: Sweden. “Jmt. Duved,” 23.07.1931, *Th. Brandt* (LD 1367491, designated by Olander and Tyler (2017: 46)).

#### Description.

Stems 25–40 cm tall, branched in the upper third, intensely purple-coloured to nearly green, completely glabrous or covered by scattered hairs 0.5–0.8 mm long. Cauline leaves 3–8 under the synflorescence, spaced, gradually decreasing towards the stem top, nearly glabrous on both sides, hairy mostly along margins. Synflorescence with long branches carrying solitary to 2–3 capitula, nearly corymbose at the top, glabrous or with solitary hairs. Phyllaries 6–7.5 mm long, purple-coloured completely or near the apex, covered by sparse hairs in the lower part or near the base, or nearly glabrous. Ray flowers dark-lilac to pale-pinkish. Pappus greyish-white.

**Figure 3.**
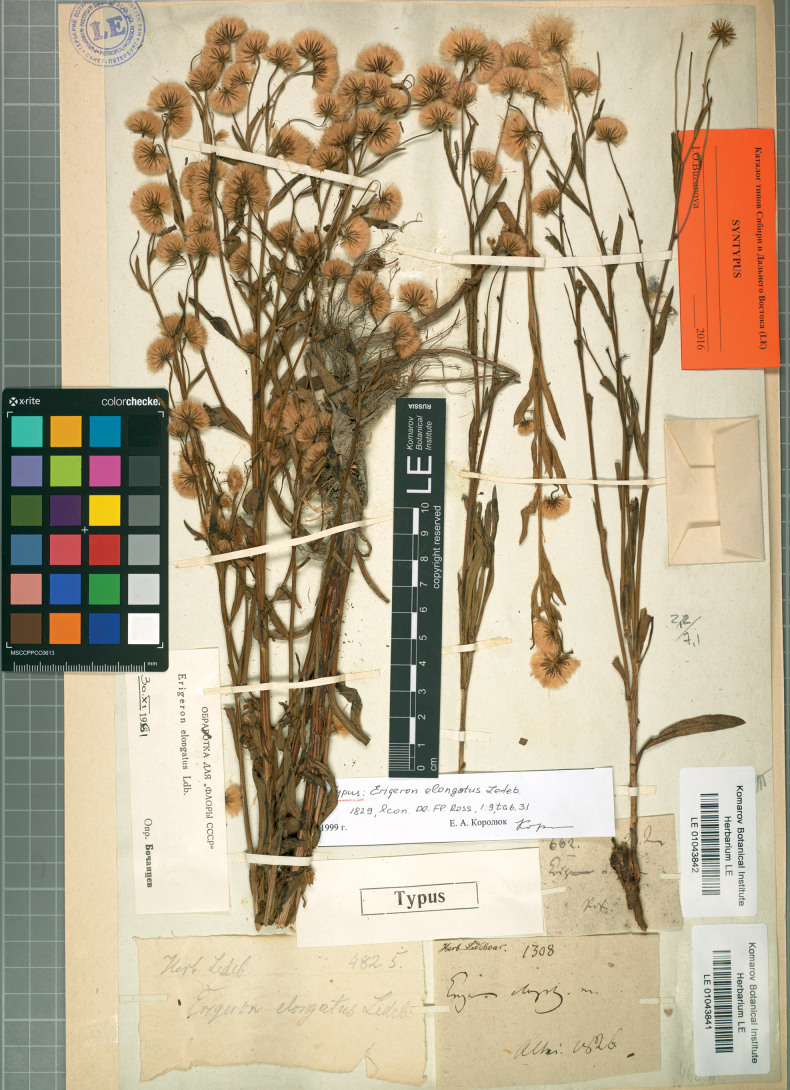
Lectotype of *Erigeronelongatus* Ledeb. (LE 1043841). Courtesy of the Komarov Botanical Institute, Russian Academy of Sciences.

Flowers in July, fruits in August.

#### Distribution in Murmansk Region.

**Figure 4.**
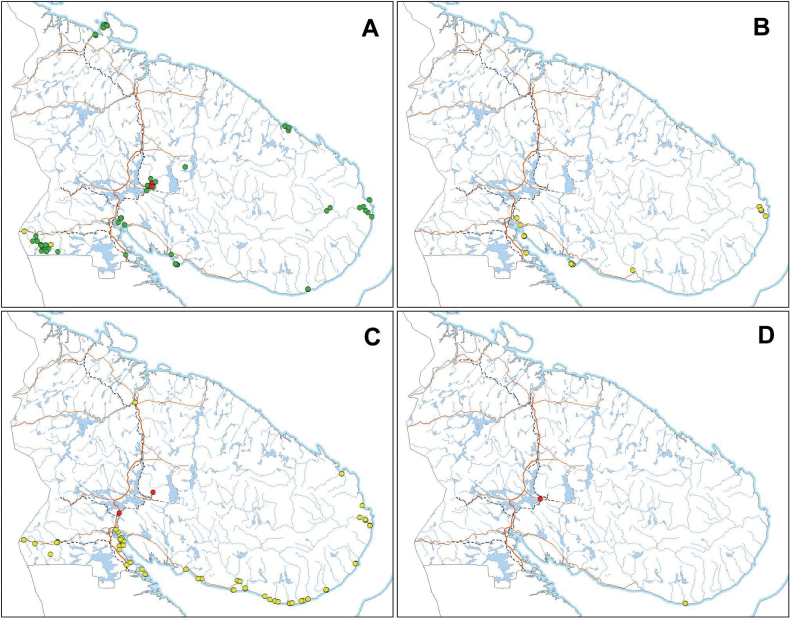
Distribution of the *Erigeronacris* group in Murmansk Region, Russia **A***E.politus* F. **B***E.×pilosiusculus* Sennikov **C***E.rigidus* Fr. **D***E.×intercalaris* Sennikov. Origin and residence status: green – native; yellow – pre-industrial alien; red – industrial alien. Maps were created using ArcGIS software by Esri. ArcGIS is the intellectual property of Esri and is used herein under license. Copyright Esri. All rights reserved.

Khibiny Mtrs., Lovozero Mts., Turii Mys, Kutsa River, Ponoi River, Orlov Cape, Kandalaksha Gulf, Rybachii Peninsula, Ambarnaya (Pikku Maattivuono) Bay, Drozdovka Village, Chapoma Village (Fig. 4A).

#### Global distribution.

Subarctic and Northern Boreal zones of Fennoscandia, Eastern Europe and Asia, mountains of southern Siberia (Altai).

#### Nomenclatural note.

The circumstances of the valid publication of *Erigeronpolitus* are rather peculiar. Fries (1843a) mentioned two variants of his *E.elongatus*, which included subglabrous, presumably perennial plants of Norwegian mountains and Lapland, of which the first one corresponded to *E.droebachiensis* and the second one was deemed to be the same as *E.glabratus* in Hooker (1834) and *E.villarsii* Bellardi in Hartman (1838). A plant of the first variant was distributed by him in his exsiccatae, “Herbarium Normale” (Fries 1842). Shortly thereafter, Fries (1843b) changed his mind and decided that the first variant (*E.droebachiensis*, which he considered the same as *E.elongatus*), was annual and the second variant was perennial, and described the latter as a new species, *E.politus*. To complement the plant distributed in “Herbarium Normale” (Fries 1842), he issued a supplement to this fascicle, which contained the only number (Fries 1843c), most probably distributed along with the protologue in August 1843. This supplement seems to be very rare in collections. Olander and Tyler (2017) were unable to trace a specimen of that gathering at S and UPS, but a good specimen is available at H. This specimen is designated here as lectotype, thus superseding the neotype designated by Olander and Tyler (2017). The lectotype has dark glabrous phyllaries 6–7 mm long, 7 capitula on long branches and much exserted ray flowers (extending the pappus up to 3 mm), and thus fully corresponds to *E.politus* as accepted in Olander and Tyler (2017).

#### Taxonomic note.

The main distribution area of *E.politus* in Murmansk Region consists of a few separate areas. Plants occurring in these areas are characterised by small but noticeable differences. Plants from the Kutsa River are exceedingly glabrous, with regularly glabrous stems and almost totally glabrous leaves, which are hairy largely along the margins; their involucres are hairy mostly at the base but forms with sparingly hairy involucre surfaces are also known. In Petsamo, plants have their involucres more regularly hairy in the basal half, and their stems are regularly but sparsely hairy. Similar plants are found in the Khibiny and Lovozero Mts., and along the Barents Sea coast. However, the plant hairiness is not completely constant, and deviating individuals can be found in all populations.

### ﻿2. Erigeron × pilosiusculus

Taxon classificationPlantaeAsteralesAsteraceae

Sennikov, sp. hybr. nov.

B35FB63A-383A-5E6B-B0B3-8250D89ADD06

urn:lsid:ipni.org:names:77331060-1

Fig. 5

#### Type.

Russia. Karelian Republic: Paanajärvi, Kauppila, torr mark nära gården [= in colle sicco], 29.07.1936, *H. Lindberg* [Plantae Finlandiae Exsiccatae no. 1369] (holotype H 039503 pro parte [plant 1]; isotypes H 339935 pro parte, OULU 059259).

**Figure 5.**
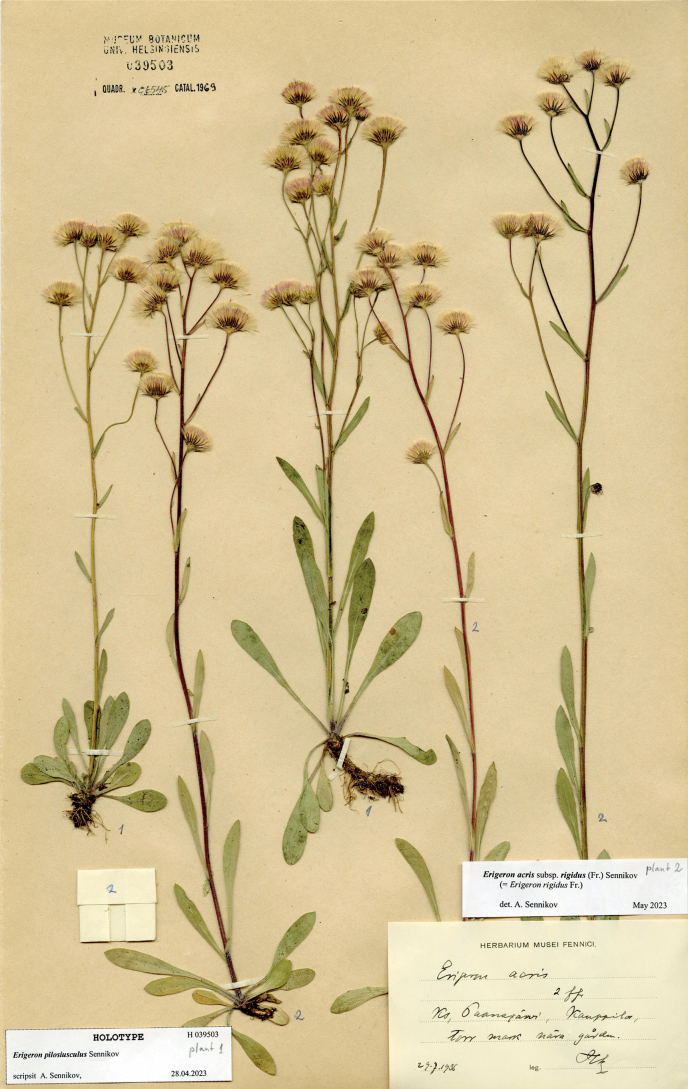
Holotype of *Erigeron×pilosiusculus* Sennikov (H 039503, plant 1). Courtesy of the Finnish Museum of Natural History, University of Helsinki.

#### Description.

Stems 25–50 cm tall, branched in the upper third, intensely to slightly purple-coloured, covered by sparse to numerous hairs 0.5–1 mm long mostly in the basal half. Cauline leaves 4–8 under the synflorescence, spaced, gradually decreasing towards the stem top, unevenly covered by sparse hairs 0.5–1 mm long on both sides. Synflorescence with long branches carrying solitary to 2–3 capitula, nearly corymbose at the top, branches subglabrous or with sparse hairs. Phyllaries 6–7.5 mm long, purple-coloured completely or near the apex, covered by sparse hairs in the basal part or up to the apex. Ray flowers dark-lilac to pale-pinkish. Pappus greyish-white.

Flowers in July, fruits in July to August. As evident from the plants collected in mixed populations, the flowering and fruiting of the hybrid occur earlier than in its native parent, *Erigeronpolitus*. When plants of *E.politus* start to blossom, the hybrid is already in the last flowers. Distribution in Murmansk Region. Kandalaksha Gulf, Turii Mys, Varzuga River (lower course), Ponoi River (lower course) (Fig. 4B).

#### Global distribution.

Subarctic and Northern Boreal zones of Fennoscandia.

#### Etymology.

The species epithet, meaning ‘slightly more hairy’ (*pilosior*, Lat.: more hairy; -*usculus*, Lat.: diminutive suffix), was selected to reflect a slighly greater hairiness of the hybrid in comparison to its more glabrous parent, *E.politus*.

#### Nomenclature note.

The type collection is taxonomically mixed. It contains typical plants of *E.rigidus* and the hybrid, which is less hairy and slightly less vigorous. This collection was distributed by Lindberg (1944) in his exsiccatae but its specimens were formed by chance: some appear to contain plants of *E.rigidus* only (H 039491), some belong only to the hybrid (OULU 059259), whereas the others may be mixed on the same sheet (H 339935).

#### Taxonomic note.

The morphology of this taxon is intermediate between *E.rigidus* and *E.politus*. Such plants typically have stems and leaves rather hairy, sometimes close to the pubescence of *E.rigidus* but never as dense and abundant as in the latter. On the other hand, its involucres highly resemble those of *E.politus* but are very sparsely covered by hairs. Because of this intermediacy, such plants were identified either as *E.politus* or as *E.rigidus*, likely depending on which part of the plant was more closely observed. We cannot refer these intermediate plants to any of the species, and therefore assume their hybrid origin, which requires a separate taxonomic placement as proposed here.

The distribution of the alleged hybrids lies completely within the area of intense anthropogenic influence, whereas only typical plants of *E.politus* were observed in the areas of its presumably native distribution (higher mountains in the centre of the Kola Peninsula and the Kutsa River basin). We consider this distribution pattern as a strong evidence for the anthropogenic origin of the presumed hybrid, which was formed within the area to which both native and alien taxa of the *E.acris* group were transported by humans.

### ﻿3. Erigeron rigidus

Taxon classificationPlantaeAsteralesAsteraceae

Fr., Novit. Fl. Suec. Mant. III: 107 (1843)

AC7AF637-8AA3-5193-B535-DDEDDD0BDE69

Fig. 6

– Erigeronacrisvar.rigidus (Fr.) A.Blytt, Norges Fl. 2: 562 (1874) – Erigeronpolitussubsp.rigidus (Fr.) Jørg. in Forh. Vidensk.-Selsk. Kristiania 1894(8): 27 (1894).
= Erigeronacrisvar.ruber Hartm., Handb. Skand. Fl., ed. 1: 315 (1820). Type. Sweden. Lule lappmark, *S.N. Casström* (holotype S, not traced).

#### Type.

Norway. Filefjell: Nystuen, *M. Blytt* (lectotype UPS, designated here).

#### Description.

Stems 25–50 cm tall, branched in the upper third, intensely to slightly purple-coloured, evenly covered by numerous hairs 0.5–1 mm long. Cauline leaves 4–8(12) under the synflorescence, spaced, gradually decreasing towards the stem top, completely covered by numerous hairs 0.5–1 mm long on both sides but subglabrous at the base below. Synflorescence with long branches carrying solitary to 2–3 capitula, nearly corymbose at the top, with numerous hairs 0.4–0.7 mm long. Phyllaries 6–7.5 mm long, purple-coloured completely or in the apical part, rather densely covered by hairs up to 0.5–0.8 mm long. Ray flowers intensely lilac. Pappus greyish-white.

**Figure 6.**
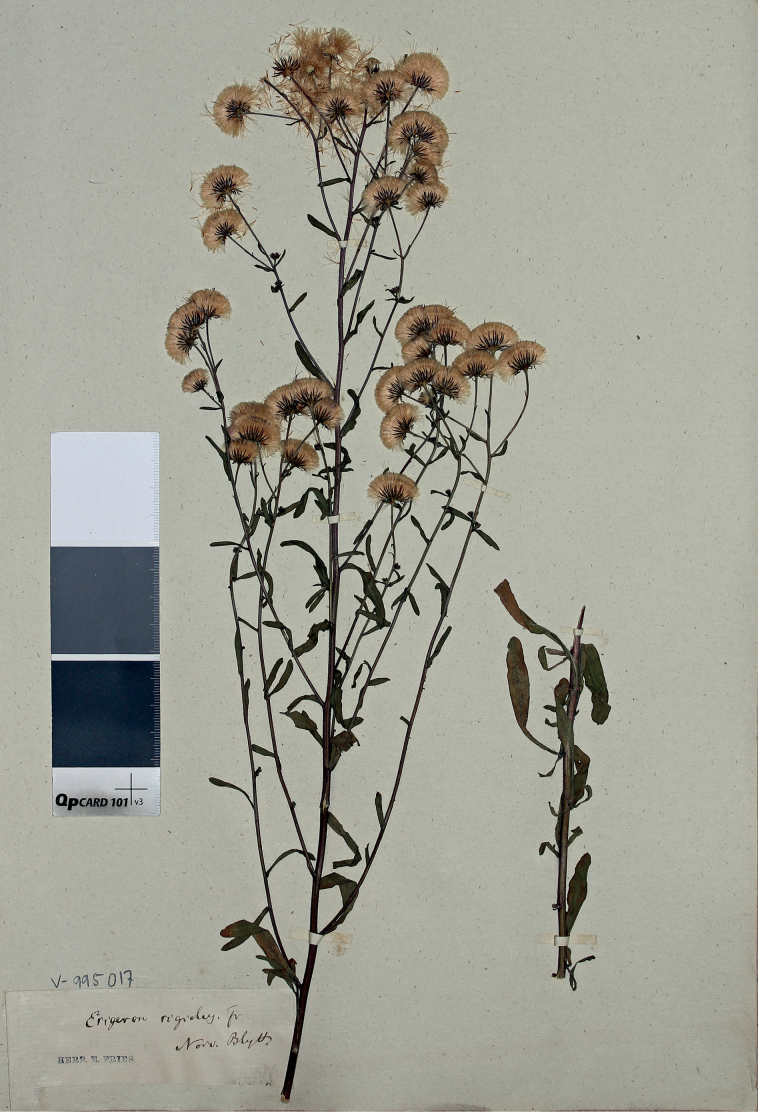
Lectotype of *Erigeronrigidus* Fr. (UPS). Courtesy of the Museum of Evolution, Uppsala University.

Flowers in July to August, fruits in August.

#### Distribution in Murmansk Region.

Coastal area of the White Sea, road from Alakurtti to Salla and Vuorijarvi Village, isolated at Kirovsk Town, Zasheyek Village and Kola Town (Fig. 4C).

#### Global distribution.

Boreal zone of Fennoscandia and Eastern Europe, southern limit unknown.

#### Nomenclature note.

Fries (1843a) mentioned two areas from which his new species was described, Filefjeld in Norway and Norrland in Sweden. The Norwegian report was based on a single specimen collected by M. Blytt in Nystuen and cited in the protologue, which is a syntype. The basis for the Swedish part of the distribution area was not specified in the protologue but Fries indicated by an exclamation mark that he had seen some (otherwise uncited) material. The specimen collected by Blytt has been traced at UPS (Hjertson, pers. comm.) and is designated as lectotype here. Hartman (1820) described Erigeronacrisvar.ruber Hartm. from Swedish Lapland, which was briefly characterised by “dark-red” ligulate flowers. This character indicates that the plant was intensely purple-coloured; together with its occurrence in Lapland, this character unambiguously points at *E.rigidus*. Quite exceptionally in those times, the protologue of E.acrisvar.ruber Hartm. (Hartman 1820) included citation of a single specimen collected by Samuel Niclas Casström, which is apparently the holotype. The collections of Casström were bequeathed after his death to the Swedish Museum of Natural History (Lindman 1916), where the holotype should be currently kept (not traced).

#### Taxonomic note.

This species is most similar to *Erigeronacris* s.str., from which it differs in typically red stems and phyllaries, and in sparser and shorter pubescence on stems, leaves and phyllaries. Its distribution area remains unknown due to the ongoing confusion with *E.acris* s.str.; so far, we feel certain to state that *E.rigidus* is common in southern Finland and Karelia, together with *E.acris* s.str., but goes farther northwards than the latter species. In Central and Southern Europe there is another similar taxon, *E.muralis* Lapeyr. (= *E.serotinus* Weihe), which apparently differs in its habit and much denser foliage ((10)17–27(40) stem leaves in *E.muralis* vs. 4–8(12) stem leaves in *E.rigidus*) (Šída 2004). Besides, *E.rigidus* flowers together with *E.acris*, whereas the flowering of *E.muralis* occurs much later (Šída 2001)

### ﻿4. Erigeron × intercalaris

Taxon classificationPlantaeAsteralesAsteraceae

Sennikov, sp. hybr. nov.

C721DEB6-F3F4-53A9-B838-577FCF4B182C

urn:lsid:ipni.org:names:77331061-1

Fig. 7

#### Type.

Russia. Karelian Republic: Louhi District, “Paanajärvi, Rajala, vägkant vid Mäntyjoki” [= northern side of Paanajärvi Lake, roadside between formerly populated places], 22.07.1936, *H. Lindberg* (holotype H 039504).

#### Description.

Stems 30–50 cm tall, branched in the upper third or half, intensely to slightly purple-coloured, evenly covered by numerous hairs 1–2 mm long. Cauline leaves 4–8 under the synflorescence, spaced, gradually decreasing towards the stem top, completely covered by numerous hairs 0.5–1(1.5) mm long on both sides or subglabrous at the base below. Synflorescence with long branches carrying solitary to 2–3 capitula, nearly corymbose at the top, with numerous hairs 0.8–1 mm long. Phyllaries 6–7.5 mm long, purple-coloured completely or in the apical part, abundantly covered by hairs 1–2 mm long. Ray flowers intensely lilac. Pappus greyish-white.

**Figure 7.**
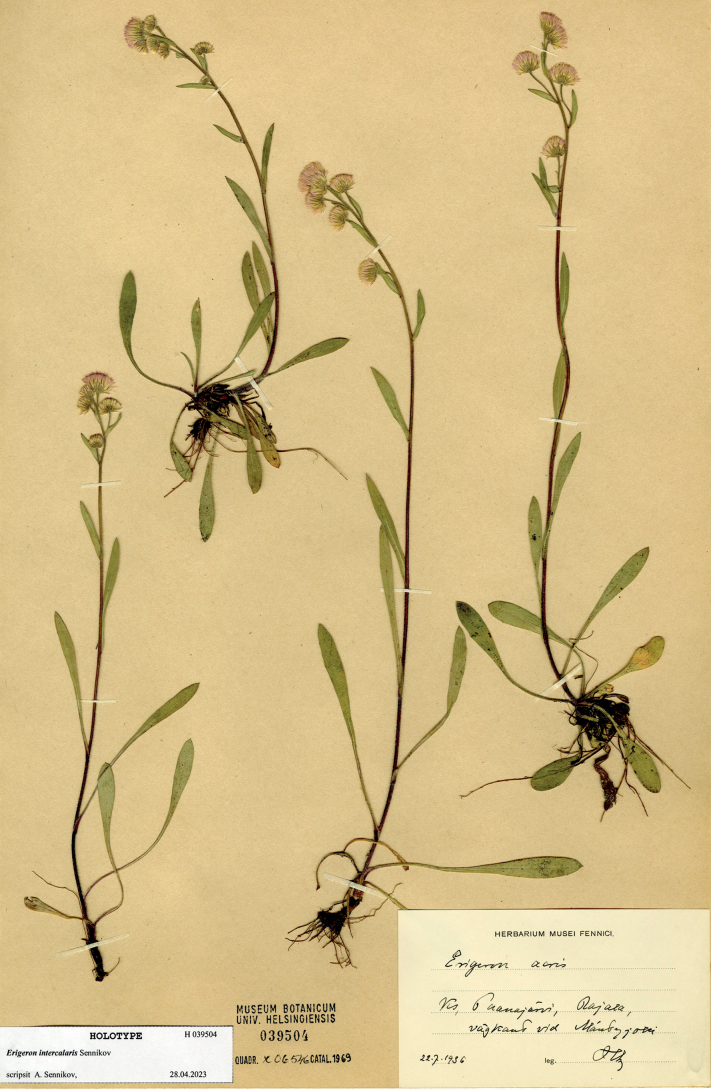
Holotype of *Erigeron×intercalaris* Sennikov (H 039504). Courtesy of the Finnish Museum of Natural History, University of Helsinki.

Flowers in July to August, fruits in August.

#### Distribution in Murmansk Region.

Tetrino Village, Apatity Town (Fig. 4D).

#### Global distribution.

Expected in the Boreal zone of Fennoscandia and Eastern Europe.

#### Etymology.

The species epithet (*intercalaris*, Lat.: intercalary) reflects the intermediate morphology of the hybrid between its presumed parents. Nomenclatural note. The type locality has been extensively sampled for *Erigeron* plants, which were distributed by Lindberg (1944) in his exsiccatae. The material from the Mäntyjoki River distributed as Plantae Finlandiae Exsiccatae 1370 is taxonomically heterogeneous: a few plants of *E.acris* s.str. (H 339936 pro parte) were mixed with abundant collections of its hybrid with *E.rigidus* (H 339936 pro parte, H 039487, OULU 059260). The latter specimens are paratypes of our *E.×intercalaris*. The hybrid plants look very slender and depressed, much less vigorous than the specimens of *E.acris* s.str. collected in the same locality, thus probably indicating outbreeding depression (Bleeker et al. 2007).

#### Taxonomic note.

The hybrid differs from *E.acris* in a regular purple colouration of its stems and phyllaries, and in a shorter and sparser pubescence on its leaves and stems. It differs from *E.rigidus* in a denser and longer hairiness of its stems, leaves and phyllaries, and in a lesser purple colouration of its stems and phyllaries.

### ﻿5. Erigeron acris

Taxon classificationPlantaeAsteralesAsteraceae

L., Sp. Pl. 2: 863 (1753).

59A6DE78-8F38-50EE-AC3A-34C1FDE11AE1

#### Type.

Probably southern Sweden. Herb. Linnaeus 994.16 (lectotype LINN, designated by Huber (1993: 44)).

#### Description.

Stems 25–40 cm tall, branched in the upper half, green or slightly to rather intensely purple-coloured, evenly covered by abundant hairs 1–1.3(1.5) mm long. Cauline leaves 5–10 under the synflorescence, spaced, gradually decreasing towards the stem top, completely covered by numerous hairs 0.5–1 mm long on both sides. Synflorescence with long branches carrying solitary to 2–3 capitula, nearly corymbose at the top, with numerous hairs 0.4–0.7(1) mm long. Phyllaries 6–7.5 mm long, green or purple-coloured on the tips, completely covered by hairs up to 0.7–1 mm long. Ray flowers pale-pink. Pappus greyish-white.

Flowers in July to August, fruits in August.

#### Distribution in Murmansk Region.

**Figure 8.**
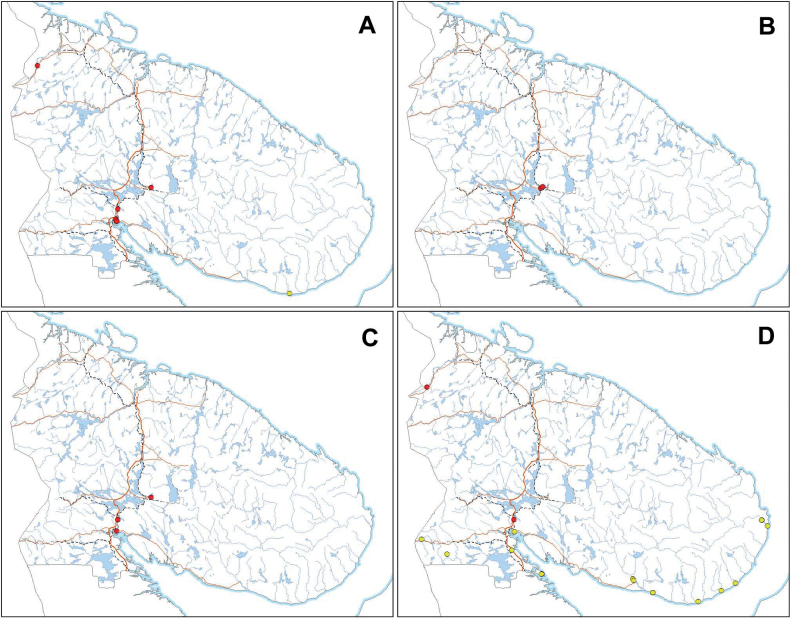
Distribution of the *Erigeronacris* group in Murmansk Region, Russia **A***E.acris* L. **B***E.droebachiensis* O.F.Müll. **C***E.uralensis* Less. **D***E.brachycephalus* H.Lindb. Origin and residence status: yellow – pre-industrial alien; red – industrial alien. Maps were created using ArcGIS software by Esri. ArcGIS is the intellectual property of Esri and is used herein under license. Copyright Esri. All rights reserved.

Kandalaksha Town, Nivsky Village, Kandalaksha and Apatity industrial areas, Apatity Town, Pasvik, Tetrino Village (Fig. 8A).

#### Global distribution.

Boreal, Hemiboreal and Temperate zones of Europe and Siberia. Nomenclatural note. The lectotype specimen at LINN was not labelled but most likely was collected by C. Linnaeus himself in Uppsala, Sweden. This specimen is a very typical representative of the species, being a greenish plant with abundant long hairs.

#### Taxonomic note.

This species is characteristic for its overall green colour of stems, leaves and phyllaries, with a red tint being present mostly at the stem base and on the tips of the phyllaries. The plant habit is the same as in *E.politus* and *E.rigidus*, with rather few sparse leaves on the stem. Another typical feature of this species is a long and dense pubescence, covering all parts of the plant (stems, leaves and phyllaries).

### ﻿6. Erigeron droebachiensis

Taxon classificationPlantaeAsteralesAsteraceae

O.F.Müll., Fl. Dan. 5(15): 4, tab. 874 (1782)

95944107-137B-5066-B6F3-FE714DCE5D01

– Erigeronacrisvar.droebachiensis (O.F.Müll.) Willd., Sp. Pl., ed. 3, 3(3): 1959 (1803) – Erigeronacrissubsp.droebachiensis (O.F.Müll.) Mela, Lyhyk. Kasvioppi Kasvio, ed. 1: 66 (1877).
= Erigeronacrisvar.angustatus Hartm., Handb. Skand. Fl., ed. 1: 315 (1820) – Erigeronacrissubsp.angustatus (Hartm.) Fr., Novit. Fl. Suec. Mant. III: 107 (1843) – Erigeronacrisf.angustatus (Hartm.) Fr., Summa Veg. Skand. 1: 183 (1846). Type. [icon] Flora Danica, tab. 874 (1782) (lectotype designated here).

#### Type.

**Figure 9.**
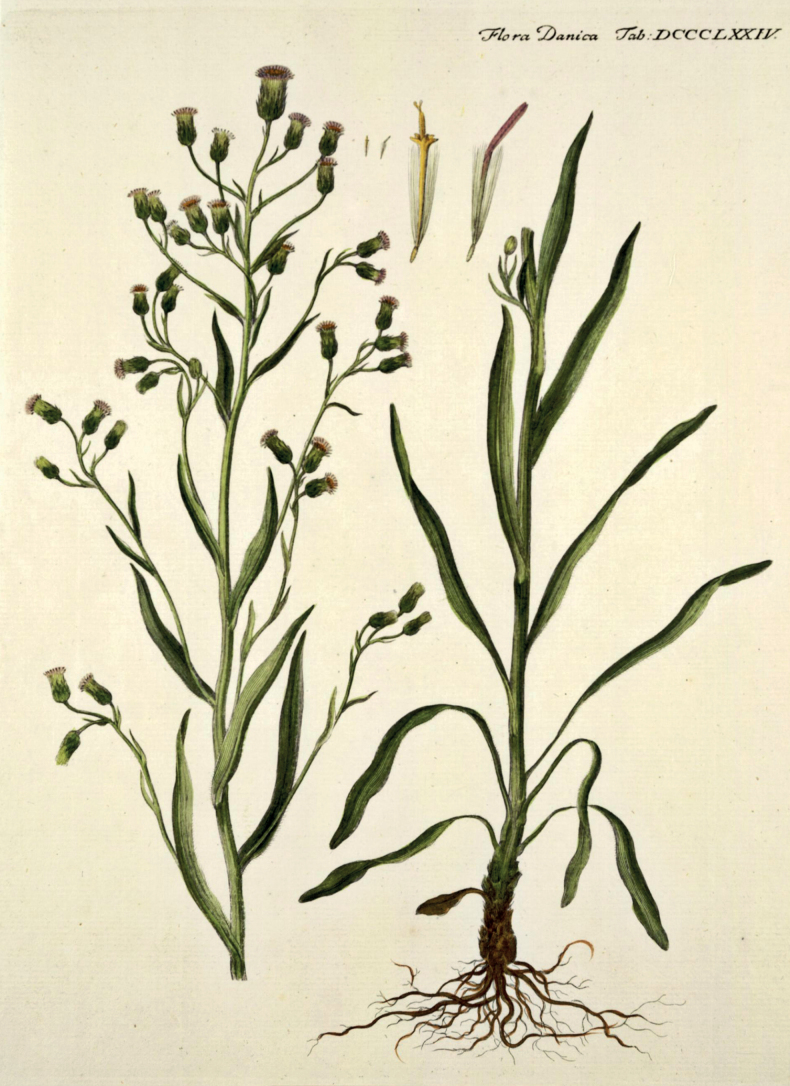
The original illustration (lectotype) of *Erigerondroebachiensis* O.F.Müll. Reproduced from Müller (1782: tab. 874).

**Figure 10.**
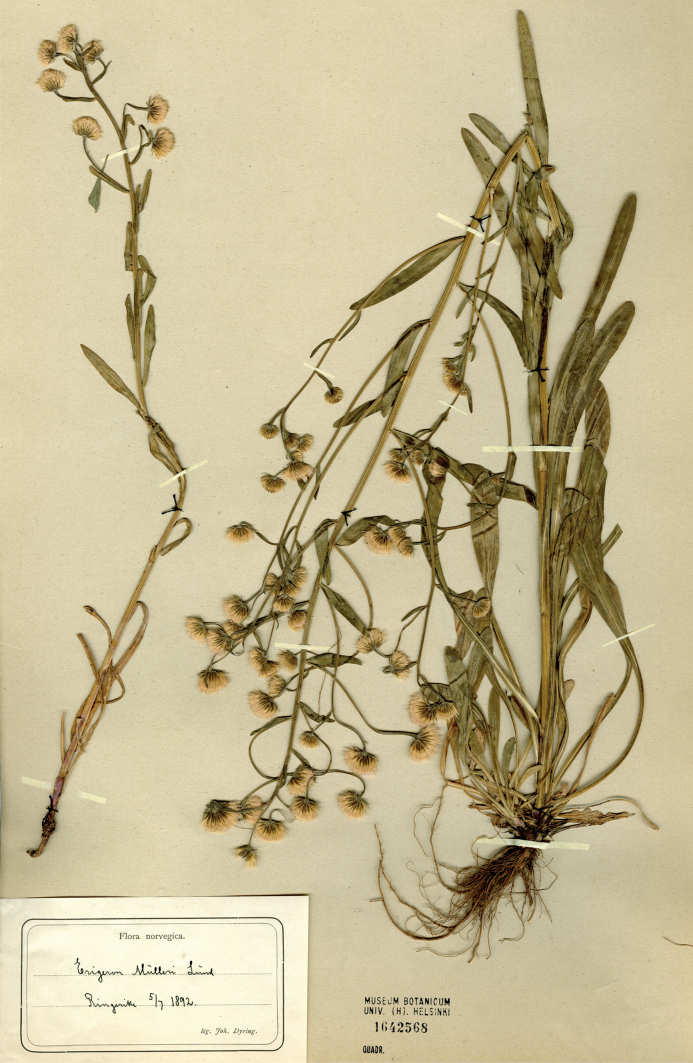
Epitype of *Erigerondroebachiensis* O.F.Müll. (H 1642568). Courtesy of the Finnish Museum of Natural History, University of Helsinki.

[icon] Flora Danica, tab. 874 (1782) (lectotype designated here). Fig. 9. Epitype (designated here): Norway. Ringerike, 05.07.1892, *J. Dyring* (H 1642568). Fig. 10.

#### Description.

Stems 30–70 cm tall, branched in the upper third, green or slightly purple-coloured, sparsely covered by numerous hairs 0.5–1 mm long in the basal third or nearly glabrous. Cauline leaves 12–20 under the synflorescence, sparse or slightly congested, gradually reduced towards the stem top, middle and lower ones covered by numerous hairs 0.3–0.8(1) mm long on both sides or along margins only. Synflorescence with rather short branches carrying few to several capitula, racemose in shape, branches glabrous or with solitary hairs 0.3–0.4 mm long. Phyllaries 5.5–6 mm long, slightly or moderately purple-coloured, outer and middle ones sparsely covered by hairs 0.5–1 mm long at base or on the basal half, innermost ones glabrous. Ray flowers pink. Pappus greyish-white.

Flowers in July, fruits in August.

#### Distribution in Murmansk Region.

Apatity industrial area (Fig. 8B).

#### Global distribution.

Boreal and Hemiboreal zones of Fennoscandia and Eastern Europe, southern limit unknown.

#### Nomenclatural note.

The species name is derived from Drøbak, now a town in Viken County, Norway, which is the original locality of the species (Müller 1782). This derivation implied the Latinisation of this place name as “Droebachia”, from which “droebachiensis” is produced by analogy with e.g. “hafniensis” that was derived from “Hafnia”, i.e. Copenhagen (Stearn 1966). The species epithet “droebachiensis” is therefore grammatically correct and cannot be changed to “droebachensis” as used in PoWO (2023), which would imply a different Latinisation as “Droebachum”. No original herbarium collections of *Erigerondroebachiensis* have been traced in Denmark (Ryding, pers. comm.) and Norway (Salvesen, pers. comm.). The only extant original element on which the species name was based is the illustration published in the protologue (Müller 1782). We agree that the original plant described by Müller was a glabrous taxon with corymbose synflorescences occurring as native in Fennoscandia, which was recognised in a similar way by other modern researchers (Tzvelev 1994; Kurtto and Väre 1998). Tzvelev (2001) attempted to radically change the application of the name *E.droebachiensis*, which he suggested to apply to a hybrid between *E.acris* s.l. and *E.canadensis* L., otherwise known as *E.×huelsenii* Vatke (Seregin 2015b). This erroneous application affected some Russian collections and literature (Seregin 2005, 2010, 2015a) but gained no recognition elsewhere. Although we agree with Olander and Tyler (2017) that the original illustration of *E.droebachiensis* unambiguously represents the species, its identity is far from apparent to those who are not familiar with the *Erigeronacris* group in Scandinavia. This is evident by the gross misinterpretation of this illustration by Tzvelev (2001), and by the uncertainty expressed by Šída (1998). To avoid further doubts and debates, we formally designate the illustration as a lectotype of *E.droebachiensis*, and support this illustration by an epitype collected in Ringerike, a traditional district situated at the distance of 50 km from Drøbak. The epitype specimen is nearly glabrous, except for the basal part of stems and capitula, and also leaf margins. A larger plant of this specimen agrees with the original illustration in a branched paniculate synflorescence, long leaves and long-exserted ligules. A smaller plant attached to the same sheet agrees with the larger plant in the pubescence and represents its reduced variant with unbranched stems, shorter leaves and a raceme-like synflorescence. Erigeronacrisvar.angustatus Hartm. was described (Hartman 1820) without any original locality indicated in the protologue. One diagnostic character of this variety (small stalked flowering heads) indicated the racemose synflorescence; the second character (larger apical capitulum) was derived from the diagnosis of *E.droebachiensis*. Subsequently Hartman (1838) explicitly noted that this variety corresponds to *E.droebachiensis*, whose illustration (but not the name itself) was cited in the protologue, and we designate this illustration as the lectotype of Hartman’s variety. Fries (1843b) elevated this variety to the subspecies level, thus creating the earliest available name at this rank.

#### Taxonomic note.

The distribution of *Erigerondroebachiensis* outside Fennoscandia is partly obscured due to its common confusion with other taxa of the *E.acris* group. Šída (1998) presumed that this species may turn to be identical to *E.macrophyllus* Herbich, which occurs in Central and Southern Europe, although the latter is characterised by more numerous and dense cauline leaves, which are 20–45 in number (Tzvelev 1994; Šída 2001).

### ﻿7. Erigeron uralensis

Taxon classificationPlantaeAsteralesAsteraceae

Less. in Linnaea 9: 186 (1834)

A6365F6A-F233-53CC-9A13-FC633504A38B

Fig. 11

– Erigeronacrisvar.microcephalus Ledeb., Fl. Ross. 2(2,6): 489 (1845).

#### Type.

Russia. Chelyabinsk Region: “Zlatoust”, 07.1832, *C.F. Lessing* (lectotype LE 01043675, designated here; isolectotype LE 01043674).

#### Description.

Stems 30–50 cm tall, branched in the upper third, intensely to slightly purple-coloured, sparsely covered by numerous hairs 0.5–0.8 mm long. Cauline leaves 8–12 under the synflorescence, sparse or slightly congested, noticeably reduced towards the stem top, very sparsely covered by numerous hairs 0.3–0.5 mm long on both sides (nearly glabrous in the middle part). Synflorescence with rather short branches carrying few to several capitula, racemose in shape, with rather sparse hairs 0.3–0.4 mm long. Phyllaries 5.5–6 mm long, slightly or moderately purple-coloured, outer and middle ones sparsely covered by hairs up to 0.5–1 mm long, innermost ones with solitary hairs. Ray flowers pink. Pappus greyish-white.

**Figure 11.**
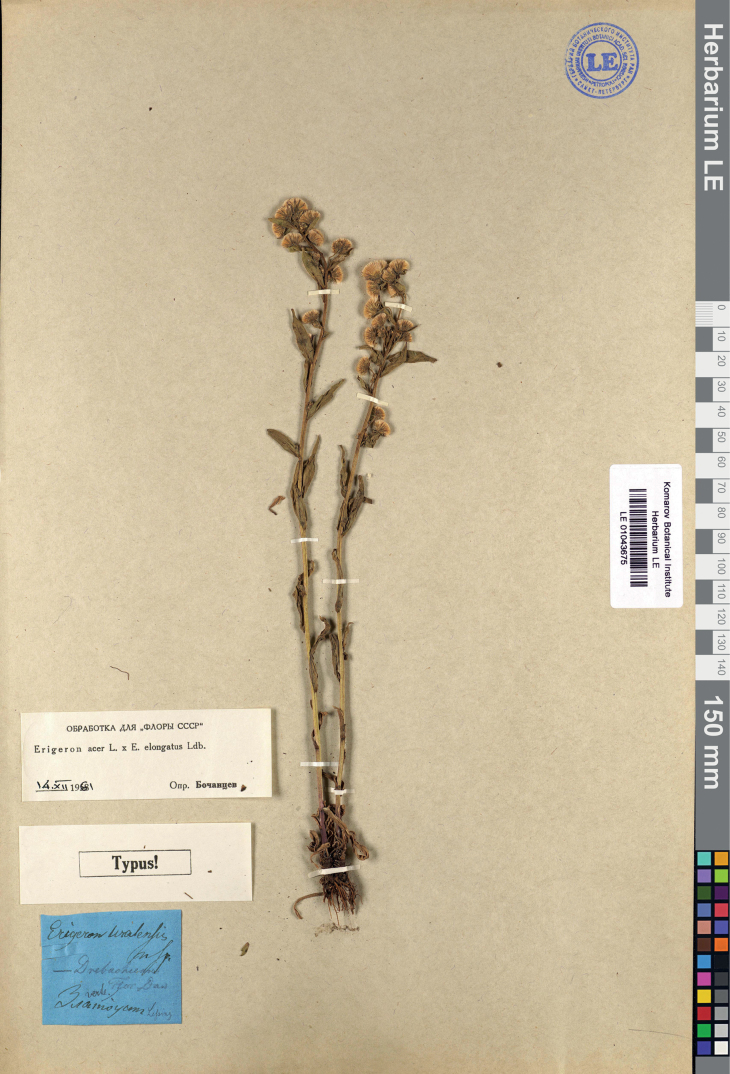
Lectotype of *Erigeronuralensis* Less. (LE 01043675). Courtesy of the Komarov Botanical Institute, Russian Academy of Sciences.

Flowers in July to August, fruits in August.

#### Distribution in Murmansk Region.

Kandalaksha and Apatity industrial areas (Fig. 8C).

#### Global distribution.

Boreal and Hemiboreal zones of Fennoscandia and Eastern Europe, Ural Mts.

#### Nomenclatural note.

The species was described on the basis of a single herbarium collection from Zlatoust Town, Chelyabinsk Region, Russia (Lessing 1834). Ledebour (1845) cited a specimen of the original collection at the Berlin Botanical Garden, which is no longer extant. Two other specimens are preserved at the Komarov Botanical Institute in Saint-Petersburg, of which one is selected here as lectotype.

#### Taxonomic note.

Tzvelev (1994) recognised a single species with numerous capitula on short branches in the Russian North, which he named *E.uralensis* and considered to include a few other previously described species. Among these synonyms, *E.decoloratus* H.Lindb. and *E.elongatiformis* Novopokr. ex Serg. were apparently added in error because they belong to the group with corymbose synflorescences (few larger heads on longer branches), whereas *E.brachycephalus* shares all essential characters with the type collection of *E.uralensis* (paniculate synflorescence with numerous heads on shorter branches, sparse pubescence on involucres and synflorescence branches). This species is seemingly distributed from Eastern Finland (Mäkelä 1980) to the Ural Mountains (Lessing 1834) and Siberia (Tzvelev 1994). Although the original material of *E.brachycephalus* largely includes specimens of *E.uralensis*, its designated lectotype (Väre 2012) differs in the density of pubescence and should be referred to another taxon. These species names are therefore not synonyms.

### ﻿8. Erigeron brachycephalus

Taxon classificationPlantaeAsteralesAsteraceae

H.Lindb., Sched. Pl. Finland. Exsicc. Fasc. 21–42: 88 (1944)

70041CF4-7ED3-5062-A6A4-23A2E6E9EB17

– Erigeronacrissubsp.brachycephalus (H.Lindb.) Hiitonen in Ann. Bot. Fenn. 8(1): 78 (1971).

#### Type.

Russia. Leningrad Region: “Isthmus Karelicus, par. Metsäpirtti [now Priozersk District], Taipale [now Solovievo], in campo sicco una cum *E.acris* (n. 1371) crescens”, 26.06.1934, *H. Lindberg* [Plantae Finlandiae Exsiccatae no. 1372] (lectotype H 340008, designated by Väre (2012: 41); isolectotype H 758234 pro parte).

#### Description.

Stems 30–50 cm tall, branched in the upper third, intensely to slightly purple-coloured, rather densely covered by numerous hairs 0.6–1 mm long. Cauline leaves 8–14 under the synflorescence, rather congested, noticeably reduced towards the stem top, completely covered by numerous hairs ca. 0.5 mm long on both sides. Synflorescence with rather short branches carrying few to several capitula, racemose in shape, with abundant hairs 0.2–0.4(0.5) mm long. Phyllaries 5.5–6 mm long, slightly or moderately purple-coloured, outer and middle ones moderately covered by hairs up to 0.5–0.8 mm long, innermost ones with sparse to rare hairs. Ray flowers bright-pink. Pappus greyish-white.

Flowers in July to August, fruits in August.

#### Distribution in Murmansk Region.

Coastal area of the White Sea, Vuorijarvi and Kuolajarvi Villages, Nivsky Village, isolated in Pasvik (Fig. 8D).

#### Global distribution.

Boreal zone of Fennoscandia and Eastern Europe, southern limit unknown.

#### Nomenclatural note.

The lectotype collection of *Erigeronbrachycephalus* is taxonomically mixed. The designated lectotype at H (Väre 2012) belongs to the more hairy taxon (*E.brachycephalus* s.str. as defined in our work), whereas its presumed duplicates at OULU and S belong to the less hairy taxon, *E.uralensis*. A duplicate at H is mixed, with both taxa mounted together. Although Lindberg (1944) described his new species as “usually” less hairy than *E.acris* s.str., by adding the word “usually” he apparently included also more hairy plants as casual variants. Further collections included into the original circumscription of *E.brachycephalus* as other syntypes belong to even more deviating taxa, e.g. *E.droebachiensis*. Although the lectotype specimen of *E.brachycephalus* is different from the other parts of this collection examined by us, it cannot be treated as incongruent with the protologue because of a broader taxonomic circumscription used by Lindberg (1944).

#### Taxonomic note.

This species is most similar to *E.uralensis*, into which it has been recently included (Tzvelev 1994). It differs from the latter in a constantly much denser and more regular pubescence on stems, synflorescence branches, leaves and involucres, and by a regular red colouration of the whole plant.

Prior to its scientific recognition, this taxon went under the collective name *E.droebachiensis* in Finland (Lindberg 1938). It was originally collected with the co-occurring *E.acris* s.str., from which it was distinguished by the paniculate synflorescence, a greater number of smaller heads, a much lesser development of pubescence and a later flowering period (Lindberg 1944).

### ﻿Identification key

**Table:** 

1	Well-developed synflorescences paniculate (compound raceme), lower leaf axils with compact raceme-like branches; phyllaries 5.5–6 mm long	**2**
–	Well-developed synflorescences corymbose, lower leaf axils with single or few capitula on long stalks; synflorescence branches glabrous or with abundant hairs; phyllaries 6–7.5 mm long	**4**
2	Synflorescence branches glabrous or with solitary hairs; outer and middle phyllaries basally or in the basal half with sparse hairs	***E.droebachiensis***
–	Synflorescence branches with sparse to abundant hairs; outer and middle phyllaries hairy up to their top	**3**
3	Synflorescence branches with abundant hairs; outer and middle phyllaries moderately covered by hairs up to 0.5–0.8 mm long	***E.brachycephalus***
–	Synflorescence branches with sparse hairs; outer and middle phyllaries sparsely covered by hairs up to 0.5–1 mm long	***E.uralensis***
4	Outer and middle phyllaries glabrous or with few hairs scattered in the basal part, inner ones without hairs; synflorescence branches glabrous or with solitary hairs; cauline leaves usually subglabrous, with hairs confined to leaf margins	***E.politus***
–	Outer and middle phyllaries with numerous hairs covering at least their basal half; synflorescence branches with numerous or abundant short hairs; cauline leaves with abundant short hairs along the whole surfaces	**5**
5	Phyllaries usually violet; outer and middle phyllaries rather densely covered by hairs up to 0.5–0.8(1) mm long; stems completely violet, with numerous hairs 0.5–1(1.5) mm long	***E.rigidus***
–	Phyllaries green, apically violet; outer and middle phyllaries completely covered by abundant hairs up to 0.7–1(1.5) mm long; stems violet at the base or in the lower half, with abundant hairs 1–1.5(2) mm long	***E.acris***

### ﻿Excluded taxa

During the whole history of botanical studies, some populations of the *Erigeronacris* group occurring in Murmansk Region were reported under wrong names.

Quite commonly taxa were treated in very broad circumscriptions; such examples are *E.acris* s.l. that included either the whole complex or its hairy representatives, or *E.politus* that included its hybrids with *E.rigidus*. Such misidentifications are too impractical to mention because of their exceedingly high number.

Sometimes, more precise identifications were published, which were mostly wrong due to vague taxonomic concepts of the past. Such identifications were rather few, and such species names are in current use for narrowly defined taxa. We traced the background for these wrong records in order to provide their correct identity (Table 1).

**Table 1.** Rejected historical records in the *Erigeronacris* group, their background and accepted identity.

Published name	Source	Basis of records	Our identification	Сomments
*E.acris* L.	Botschantzev 1959	LE 01102450,	E.×intercalaris	these specimens are very similar to *E.acris* s.str.
		LE 01102451		
*E.acris* L.	Orlova 1966	many specimens at KPABG	mostly *E.rigidus* and its hybrids, *E.brachycephalus*, one specimen of *E.acris* s.str.	including all hairy taxa of *E.acris* s.l.
*E.acris* L.	Tzvelev 1994	LE 01102456	*E.rigidus*	also the material used in Botschantzev (1959)
*E.acris* L.	Ulvinen 1996	OULU 158562	*E.rigidus*	*E.acris* s.str. is absent in this territory
*E.acris* L.	Kravchenko 2020	TROM 54773	*E.brachycephalus*	his second record belongs to *E.acris* s.str.
*E.decoloratus* H.Lindb.	Ulvinen 1996	H 039483	*E.politus*	mere misidentification
		H 039484		
*E.droebachiensis* O.F.Müll.	Fellman 1864	H 846840	*E.brachycephalus*	plants with racemose synflorescences
		LE 01102478		
*E.droebachiensis* O.F.Müll.	Mäkelä 1980	H 340138	*E.rigidus*	also the material at H used by Fellman (1864)
		H 340141		
		H 340142		
		H 340146		
*E.uralensis* Less.	Tzvelev 1994	LE 01102459	*E.politus*	slender plant with longer branches

## ﻿Discussion

### ﻿Diagnostic characters, their value and variability

Although we relied upon diagnostic characters on which the previous works (Tzvelev 1994; Kurtto and Väre 1998; Šída 1998, 2000; Olander and Tyler 2017) have been based, every character was reassessed in the course of our revision.

Easy to catch is the feature of purple colouration, which may affect all vegetative parts of the plant: stems, leaves and phyllaries. We found this character to be of good subsidiary value: although it may quite widely vary in plants of the same taxon being nearly green to completely purplish (e.g. *E.politus*), it may reliably serve for primary diagnostics between *E.rigidus* (purplish) and *E.acris* s.str. (green).

Synflorescence shape (corymbose vs. racemose) is found to be a strong and highly reliable character, in agreement with the work of Šída (1998, 2000, 2004). In weak plants, synflorescences may be reduced and their shape may appear uncertain; in such cases, this character can be inferred from the length of pedicels (synflorescence branches) and the number of capitula, as already used by some researchers (Tzvelev 1994; Kurtto and Väre 1998).

Size of capitula, measured as length of phyllaries, is an important character apparently correlating with the synflorescence shape. It can be used as a proxy for the latter, too.

Number, shape and density of cauline leaves have been commonly used to distinguish between some taxa in Central and southern Eastern Europe (Tzvelev 1994; Šída 1998, 2000, 2004), which may differ in the density of foliage and ultimately the absolute number of stem leaves below the synflorescence. Although these characters are undoubtedly useful in the *E.acris* group, their use in the European North is rather limited because of the lack of the densely leaved taxa. In general, our plants with corymbose and racemose synflorescences may differ in the number of cauline leaves and their density, but this difference is not so prominent and this character can be used as auxiliary here.

Flower colour (ligulate flowers) varies between pale and dark lilac, rarely (in *E.decoloratus*) white ligules were observed in plants outside the study area. We noticed that this character correlates with the purple colouration of stems and phyllaries and is therefore similarly variable, and sometimes may vary within a single plant when one branch is purplish and the other is greenish. For this reason we do not give a separate diagnostic value to this character.

Length of ligulate flowers was sometimes used (Fries 1846; Olander and Tyler 2017) but we found this character variable within the same taxon. Its value is rather uncertain.

**Figure 12.**
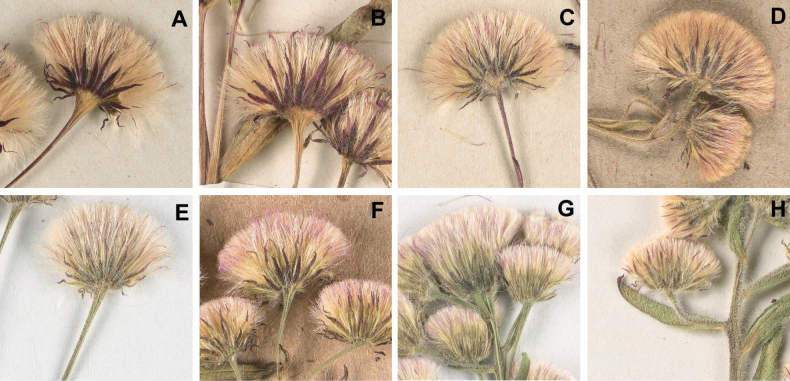
Flowering heads of the *Erigeronacris* group in Murmansk Region **A***E.politus* Fr. (KPABG 040062) **B***E.×pilosiusculus* Sennikov (KPABG 039993) **C***E.rigidus* Fr. (KPABG 040017) **D***E.×intercalaris* Sennikov (KPABG 039998) **E***E.acris* L. (KPABG 043965) **F***E.droebachiensis* O.F.Müll. (KPABG 047662) **G***E.uralensis* Less. (KPABG 043994) **H***E.brachycephalus* H.Lindb. (KPABG 040025).

Pubescence (presence or absence of simple hairs) is considered another primary taxonomic character (Tzvelev 1994; Kurtto and Väre 1998; Šída 1998, 2000; Olander and Tyler 2017). Plants in the *E.acris* group differ remarkably in the density and length of pubescence on stems, leaves and phyllaries. Despite a certain level of variability, we find this character reliable in distinguishing taxa within the groups with different types of synflorescence, with the hairiness ranging from extremely sparse (*E.politus*, *E.droebachiensis*) through moderate (*E.rigidus*, *E.uralensis*) to abundant (*E.acris* s.str., *E.brachycephalus*). The length of pubescence is important to help distinguishing between some taxa with shorter (*E.rigidus*, *E.brachycephalus*) and longer (*E.acris* s.str.) hairs, and can be used when the other characters are expressed ambiguously. The differences in pubescence are most easily observable on the involucres (Fig. 12).

Achene characters are difficult to use in herbarium specimens, which are collected mostly in flower (Olander and Tyler 2017). However, we noticed that achene hairiness may vary within the same narrowly defined taxon, and the diagnostic value of this character is therefore doubtful.

### ﻿Comparisons of taxonomic concepts

The first attempt to classify the diversity of the *Erigeronacris* group was made by Müller (1782) who described *E.droebachiensis* from a single locality in Viken County, Norway. This species was separated not because of the advanced knowledge in this taxonomic group; instead, it was compared with *E.canadensis* L., a distantly related species which appears to be only superficially similar because of its racemose synflorescence.

Early botanical works recognised only a single species in the *E.acris* group, but its apparent morphological variability was reflected in varieties. Hartman (1820) noted some differences in the size of flowering capitula and the colour of ligulate flowers as taxonomically significant characters; at the same time, he merged the previously described *E.droebachiensis* even without a note on its name.

Elias Fries (1843a, 1843b, 1846) recognised several taxa in this group, at the level of species and below. He used several characters to ground his taxonomy, including synflorescence shape, size of capitula, features of foliage, pubescence of all parts of the plants, ligulate flower colour and length. Most notably, he distinguished between the southern *E.acris* s.str. with an abundant soft pubescence and the northern *E.rigidus* with sparser stiff hairs, the distinction being of a wide phytogeographic importance but commonly neglected in later taxonomic works.

Fellman (1864) reported *E.droebachiensis* from Russian Lapland; however, this was not the taxon in our current understanding.

Mela (1877, 1884, 1895, 1899) developed a complicated taxonomic classification for the *E.acris* group in Finland, in which he recognised 10 infraspecific taxa at the level of subspecies, variety, subvariety or forma. His primary distinction was laid between *E.acris* s.l. and *E.droebachiensis* s.l., which he distinguished on the basis of hairiness and subdivided further for the size and colour of capitula and minor details of pubescence. The two-taxon system in the *E.acris* group developed by Mela was used in contemporary Finnish publications (e.g., Saelan et al. 1889). His elevation of taxonomic ranks in this group (Mela 1895, 1899), giving the species status to *E.droebachiensis*, was not supported by other botanists.

Botschantzev (1959) attempted to revise the *E.acris* group in the USSR. He recognised only two taxa in the European North, the glabrous *E.politus* and the hairy *E.acris*, between which abundant hybrids were allegedly found. His treatment was based on the collections at LE. The first detailed taxonomic revision of this group in Murmansk Region was provided by Orlova (1966), who followed Botschantzev (1959) in the taxonomy. Orlova revised the collections at KPABG and provided point distribution maps of both taxa accepted in the territory.

Tzvelev (1994) provided a new taxonomic treatment of the *E.acris* group in the European part of the USSR. In the North, he maintained the two taxa recognised by his predecessors in the same circumscription but added the ill-defined collective species *E.uralensis*, which he distinguished by its numerous flowering heads (paniculate synflorescence) and believed to have originated from interspecific hybridisation between *E.politus* and *E.acris*. He collected three synonyms under this species name: *E.brachycephalus*, *E.decoloratus* and *E.elongatiformis*. By doing so, Tzvelev disregarded the character of paniculate vs. corymbose synflorescences (*E.brachycephalus* and *E.uralensis* have paniculate synflorescences, whereas *E.decoloratus* and *E.elongatiformis* have corymbose synflorescences) and apparent differences in pubescence. He used many diagnostic characters to distinguish between his species, and the number of taxa that he accepted was the highest among all taxonomic revisions to date. Nevertheless, the inconsistent use of morphological characters in his treatment did not allow him to achieve a clear picture of plant taxonomy and distributions.

The latest Finnish synopsis of the *E.acris* group (Kurtto and Väre 1998) was most detailed in Eastern Europe. It accepted five subspecies of *E.acris* s.l. on the basis of pubescence, presence or absence of a purple tint, and number of flowering heads. The subspecies were assessed according to their residence status, as native or alien (archaeophyte), and mapped according to traditional biogeographic provinces of East Fennoscandia, thus revealing their distribution patterns. This treatment was quite fairly and accurately set, except for the broad treatment of E.acrissubsp.acris (which included *E.rigidus*) and the lack of recognition of hybrids.

Synflorescence shape was considered a primary character by Šída (1998, 2000, 2004), who based the uppermost-level division in his classification (ranked as series: E.ser.Trimorpha (Cass.) Šída, E.ser.Macrophylli Šída) on this character but also added a separate series (E.ser.Politi Šída) to accommodate completely glabrous plants. The main classification rank in this system was species. Šída provided a brief overview of the *E.acris* group in the whole of Eurasia (Šída 1998) and a detailed taxonomic treatment for the Czech Republic (Šída 2000, 2004). He precisely defined the species and mentioned the presence of hybrids. His work provided highly useful insights for our treatment but could not be used at the species level.

The latest taxonomic revision of the *E.acris* group in Sweden, with taxonomic implications for Fennoscandia (Olander and Tyler 2017), was based on a complex statistical analysis of numerous measures rather than traditional observations. Olander and Tyler (2017) believed that their accepted taxa (ranked as subspecies) may be distinguished by a combination of the characters of pubescence and dimensions (of stems, leaves and phyllaries, also including leaf and capitula number). However, they failed to observe minute differences in pubescence between *E.rigidus* and *E.acris*, and also reduced *E.brachycephalus* to a synonym of *E.droebachiensis* despite their apparently different types of pubescence, probably because they had insufficient material for examination.

### ﻿Phenology

The available herbarium collections indicate that flowering period may be highly variable due to the differences in particular season, vegetation zone or even local conditions. In the same locality but in different years, plants of the same species may start flowering with a difference as high as a month. At the same time, plants collected within one day in the same place may show differences corresponding to one week of observations within a single population. Plants collected in different vegetation zones (tundra vs. taiga) may start flowering with a delay of two weeks or even greater, whereas the flowering period may be quite short and limited to two or three weeks. This makes summarily observations within the whole territory of Murmansk Region practically meaningless.

At the same time, plants of different taxa may develop in clearly different periods when observed as co-occurring within the same locality. In this case, plants of *E.rigidus* and its hybrids may develop significantly earlier than those of *E.politus*, whereas plants of *E.brachycephalus* start flowering apparently later than those of *E.rigidus*. Since in such cases the difference in flowering periods is approximately a week or less, the summary observations may practically coincide.

### ﻿Geographical distributions

Distribution patterns of each species in the *Erigeronacris* group are individual. Although some species may co-occur in the same locality and may share some part of their history of dispersal, their main sources and drivers seem to be different.

Due to a considerable confusion between the segregate taxa in this group even in the most detailed treatments (Mäkelä 1980; Tzvelev 1994), their major distribution areas remain partly obscure. The lack of separation between the native and secondary parts of distribution areas in these works makes the identification of their origin even more difficult.

*Erigeronpolitus* is a native taxon, which occurs in mountains and uplands, and in some fjords and river ravines along sea coasts in Murmansk Region. This species is a largely subarctic (oroarctic) plant in Fennoscandia (Mossberg and Stenberg 2018). Its secondary dispersal is very minor and limited to short-distance transfer of diaspores from native populations to neighbouring anthropogenous habitats (roadsides, waste lands, populated places). The species is favoured by disturbance and can be found growing rather abundantly in industrial waste lands.

**Figure 13.**
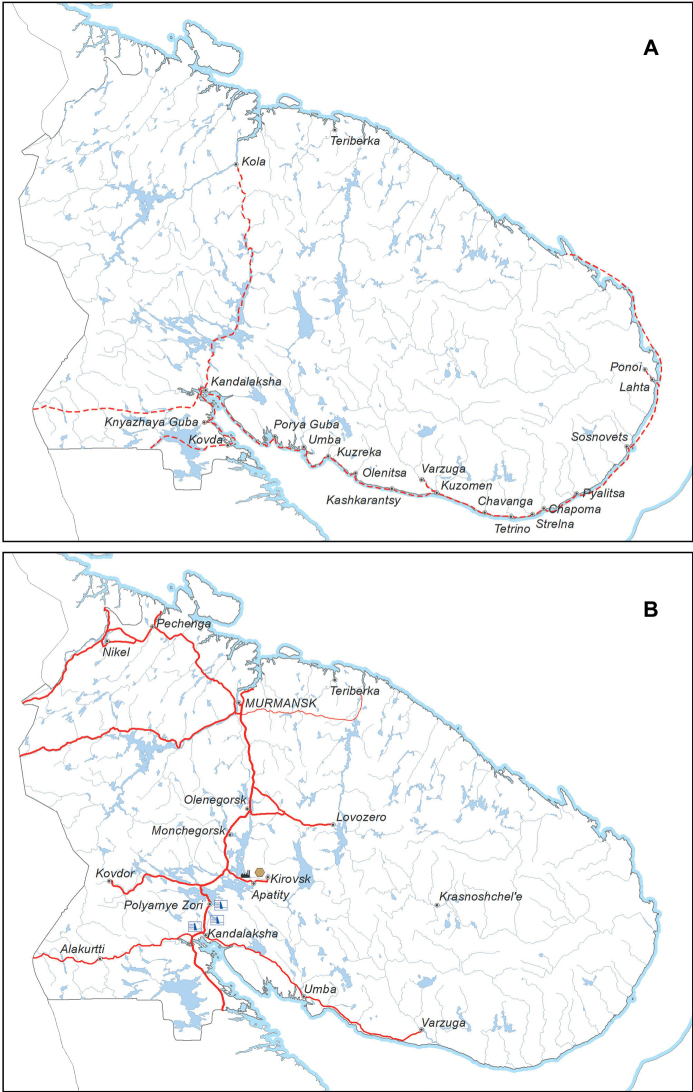
Main transport networks and their major populated places in Murmansk Region **A** pre-industrial period **B** industrial period. Red dashed lines (**A**) show the Pomors transport route along the White Sea, the Kola road to the north, and two trade communication roads between the White Sea and northern Finland. Red solid lines (**B**) indicate main roads, black dashed lines (**B**) are railways. Special signs (**B**) denote electric thermal (black) and hydro (blue) power stations, and nepheline mining area (brown) mentioned in the text. Maps were created using ArcGIS software by Esri. ArcGIS is the intellectual property of Esri and is used herein under license. Copyright Esri. All rights reserved.

The present-day distribution of *E.rigidus* in Murmansk Region seems to be a fair reflection of its historical dispersal. The species distribution is limited to two major areas: the entire White Sea coast with adjacent islands, which was a traditional area of the Pomors economy, and the Alakurtti–Salla road, which was a historical traffic route between northern Russia and Finland (Fig. 13A). Two isolated localities can be explained by further dispersal: Kola (by historical trade) and Apatity (more recent dispersal in the early industrial times, but most likely with some local and traditional-like traffic). In undoubtedly native habitats (tundra and remote river ravines, in which *E.politus* typically occurs) this species is always lacking.

The distribution of *E.brachycephalus* follows the same pattern but is much sparser, also suggesting its connection with the pomors. However, two of its known localities have a recent origin. In the Pasvik area, the species was collected from a recently abandoned Russian military camp (erroneously reported as *E.acris*: Kravchenko 2020), which had a post-war origin, whereas its presence along the channel of a hydroelectric power station may be linked with revegetation activities during the late Soviet times.

Despite the broad occurrence erroneously reported in the past, *E.acris* s.str. was found only in a few scattered localities, mostly very recently. The only old locality of this species is known at Tetrino, along the southern coast of the Kola Peninsula. This population is part of a local hybrid swarm, found together with *E.rigidus* and their hybrids in 1937, and therefore can also be linked with traditional activities of the pomors.

A large area of the recent introduction of *E.acris* is situated along the Niva cascade of hydroelectric power stations (Fig. 13B). This cascade continues from Kandalaksha Town in the south to Lake Imandra in the north. To date, plants of *E.acris* were recorded in connection with the second and third power stations, growing in large populations. Another isolated population was found on abandoned stockyards of an apatite-nepheline processing plant (concentrating mill) (Fig. 13B). One more locality, in Pasvik (Kravchenko 2020), is linked with another abandoned military place.

*Erigeronuralensis* was newly discovered in three localities along the Niva cascade of hydroelectric power stations and on abandoned stockyards of an apatite-nepheline processing plant. It was found in mixed populations together with *E.acris*.

*Erigerondroebachiensis* was collected twice in a single locality, an abandoned slag-dump at the Kirovsk thermal electric power station (Fig. 13B). This place of introduction is not connected with any other taxon of the *E.acris* group, thus showing its independent origin.

### ﻿Pathways and periods of introduction of alien species

It may be exceedingly difficult to establish pathways and periods of introduction of particular alien plants in the territories with a long and complicated history of introductions, or even to distinguish between native and alien plants. However, the low natural floristic richness of the Arctic is particularly helpful in revealing alien plants, and its harsh climatic conditions efficiently limit the introduction and further spread of alien plants in this territory; for this reason, the diversity of non-native alien plants in the Arctic is still considerably lower than at the southern latitudes (Wasowicz et al. 2019).

The territory of Murmansk Region belongs partly to the Subarctic tundra, partly to the Northern Boreal forest (Chernov 1971). These species-poor landscapes clearly allow for detecting alien plants when present outside seminatural or human-transformed landscapes near inhabited places or places of economic activities. We considered habitats and distribution patterns of taxa of the *E.acris* group in Murmansk Region and determined that only one species, *E.politus*, is undoubtedly native in the territory because its occurrence is predominantly linked to native landscapes, whereas the other taxa are clearly alien because they are confined to the areas of historical or modern human activities. This conclusion provides a major correction to all previous treatments of this group in Murmansk Region (Botschantzev 1959; Orlova 1966; Tzvelev 1994), which were based on the belief that ‘*E.acris*’ (i.e. the *E.acris* group excluding *E.politus*) is also native to this territory due to its wide distribution and common occurrence in seminatural habitats. However, the occurrence of *E.rigidus* and *E.brachycephalus* is confined to the areas with strong anthropogenous influence; their localities are typically situated nearby old human settlements or places of traditional occupational activities (fishing, saltmaking, mining).The earliest records of *E.rigidus*, documented by herbarium specimens at H, were made in the 19^th^ century near old Russian villages along the southern coast of the Kola Peninsula and along the lower course of the Ponoi River and Kola River, as well as in several Finnish villages along the road from the White Sea to Salla via Alakurtti (formerly in Oulu or Lapland Region, Finland) which was situated on a major communication road between the Russian and Finnish North (Ahti and Hämet-Ahti 1971). These records evidenced that in the 19^th^ century the species had been firmly established and widespread in the territory, and already connected with the areas of traditional Russian economic activities. Its more precise period of introduction to the territory can be only inferred from its historical occurrences: some older Russian villages along the southern coasts were established well before the 16^th^ century, which is the accepted chronological limit between archaeophytes and neophytes (Pyšek et al. 2002), and the traditional Russian economy along the southern coast of the Kola Peninsula has continued since the 12^th^ century (Bernstam 1978). As the factors that can be linked to the present-day distribution of *E.rigidus* in the Kola Peninsula (economic activities of the Pomors) emerged and became strong so early in the history, we conclude that this species is most likely an archaeophyte in Murmansk Region.

The pathways of introduction of *E.rigidus* are linked to the economic activities of the Pomors. The taxa of the *E.acris* group are very minor and insignificant weeds of field crops (Krascheninnikov et al. 1935), and their effective dispersal as contaminants is unknown and therefore unlikely. Furthermore, the species occurrence in Murmansk Region strongly suggests that its local dispersal was not directly linked with transport of goods or commodities like grain; on the contrary, the species is very commonly found on fishing and sailing places in which no cargo had been discharged, with the continuous occurrence along the coasts. For this reason, we assume that the species was introduced and further dispersed adhering to clothes, footwear and other items possessed and moved by travellers, who may be trading, fishing or performing any other traditional occupation. To the Kuusamo area, it may have been introduced by Russian peddlers who were known to trade regularly in the area (Ahti and Hämet-Ahti 1971). This pathway can be classified as Transport-Stowaway: People and their luggage/equipment (Harrower et al. 2018). Local colonisation occurred not only by wind; it was apparently aided by cattle (lambs and cows), as evident from historical records on pastures and along small watercourses that are known to have been used for grazing (Zaitseva 2000).

The historical occurrence of *E.brachycephalus*, which is similar to the distribution of *E.rigidus* but much sparser, suggests that this species was introduced and dispersed using the same agents and factors but likely in later times, probably in the 16^th^–17^th^ centuries when further large villages were established in the Kandalaksha Gulf and along the lower course of the Ponoi River (Bernstam 1978). This species can therefore be classified as an early neophyte. Its distribution was formed using the same pathway and source of introduction as those that shaped the distribution of *E.rigidus*.

Bernstam (1978) suggested that the Pomors originated in the Ladoga area, southern Karelia, from which they gradually colonised the coasts of the White Sea. Their origin and vector of colonisation agrees with the nearest native distribution area of *E.rigidus* and *E.brachycephalus*, which we define as central and southern Karelia. In northern Karelia, these two species were introduced by humans many hundred years ago.

Since historical records of *E.acris* are limited to a single village (Tetrino) without further localities along the sea coast, we assume this occurrence to have originated from old long-distance dispersal. Tetrino is an early village on the coast, dated from the 17^th^ century, and its history was connected with the Resurrection Monastery at Istra (now Moscow Region) that established the village (Bernstam 1978) and the Solovetsky Monastery that, before the revolution, in the 15^th^–18^th^ centuries possessed and in the 18^th^–20^th^ centuries supervised territories of the former Tetrino District (Dositheos 1836). The monastery traffic from the Solovetsky Archipelago (now Archangelsk Region) may have been responsible for the long-distance dispersal of *E.acris* and its introduction to the territory as an early neophyte.

The modern introduction of *E.brachycephalus* and *E.acris* to military camps is linked with longer-distance transportation but probably the same pathway of introduction; it occurred in the late Soviet period after the Second World War. Similarly, cargo traffic was responsible for the recent occurrence of *E.acris* at the Apatity railway station. So far, it is uncertain what kind of item was contaminated with the *Erigeron* seeds.

*Erigeronacris* and *E.uralensis* were collected on abandoned stockyards of the First apatite-nepheline processing plant (concentrating mill) of the Apatite mining and processing enterprise. These stockyards functioned during 1956–1963 (Gershenkop et al. 2010; Mazukhina 2019) to accumulate and dump tailings produced while processing apatite-nepheline ore. After this period, the stockyards were closed and their territory was revegetated (covered by ground and plant cover), in agreement with the standards (Smetanin 2000; Chibrik 2002). To achieve a higher density of the restored plant cover, seeds of rhizomatous perennial herbs and grasses have been used since 1964 (Kryuchkov 1985; Druzhinina and Mialo 1990; Timofeeva et al. 2016). Such seeds were commercially produced in the USSR during the revegetation period rather than imported from abroad (Zolotarev et al. 2017). This type of revegetation was standard for all types of industrial lands, including slag-dumps (Smetanin 2000; Belozerova 2006). When similarly revegetated slag-dumps of the Ural Region were examined botanically, the high presence of *E.acris* s.l. was noted as a weed, especially on bare ground patches (Glazyrina et al. 2009; Chaschikhina 2021). This presence can be explained by contamination of the seed used in revegetation, also in Murmansk Region. This pathway can be classified as Transport-Contaminant: Seed contaminant. The source territory of introduction should be situated at least at Saint-Petersburg or farther southwards, where both alien species may co-occur. A more remote location of the source area may be indicated by the fact that *E.rigidus*, which belongs to the most common taxa in southern Karelia and Leningrad Region, has never been found on revegetated grounds.

We also considered a possibility for the seeds of the *E.acris* group to arrive with contaminated soils and found this pathway practically impossible. The topsoil used for revegetation in Murmansk Region was locally excavated peat rather than any substrate imported from previously vegetated places (Kryuchkov 1985). Although many ruderal plants, including the *E.acris* group, were observed on revegetated grounds in Krasnoyarsk Region where fertile soil had been used in revegetation (Efimov and Shishikin 2014), local peat can be considered completely free of any unwanted botanical contamination.

An abandoned slag-dump at the Kirovsk thermal electric power station, on which the only locality of *E.droebachiensis* was found, has been revegetated in a similar way but after 1990 (Davydov and Redkina 2021). The nearest distribution area of this alien taxon is Southern Karelia, where it occurs together with *E.uralensis* and *E.brachycephalus*.

The same two taxa, *E.acris* and *E.uralensis* (together with *E.brachycephalus*), were found along the Niva cascade of hydroelectric power stations (HPS). The last power station in this cascade was completed in 1954. This construction suggests that revegetation on the channels and dams of this cascade should have occurred no later than in the 1960s. The alien species composition and their presumed period of introduction essentially coincide for the cascade of HPSs and the mining stockyards, thus indicating a likely similar origin of the seed used in revegetation in both cases.

The occurrence of *E.acris* and *E.uralensis* at the mouth of the Niva River suggests their self-dispersal downstream from the places of their original introduction. This possibility is confirmed by the experiments indicating the ability of seeds of the *E.acris* group to drift along watercourses and remain viable in the end (Bill et al. 1999).

### ﻿Naturalisation and further spread of alien taxa

All the non-native taxa of the *Erigeronacris* group found in Murmansk Region can be considered naturalised aliens. These plants are biennial or short-lived perennial, reproducing by seed, and self-sustaining populations are essential for their continuous presence in the territory, which has been repeatedly observed by numerous collectors in various localities.

From the sparsely scattered pattern of historical records of *E.acris* s.str. and *E.brachycephalus* and the continuous distribution of *E.rigidus*, all having been introduced to Murmansk Region with travelling humans but in different time periods, we infer that some kind of longer-distance dispersal may have occurred to deliver the first propagules of these species to the territory. This introduction was subsequently complemented by short-distance dispersal with the same agents and by local dispersal with cattle.

In spite of the long period of introduction, none of these tree taxa became truly invasive. All these species formed stable local populations in semi-natural landscapes or near populated places, but none of them shows a tendency to expand from their locally restricted refugia further into native landscapes.

The alien populations introduced in the post-industrialisation period (after the Second World War) were recorded in technogenic landscapes or in military areas. In most cases these alien plants were not observed outside the area of their original introduction. However, the occurrence of *E.acris* and *E.uralensis* along the Niva cascade of hydroelectric power stations demonstrates their potential for further dispersal by running water and by wind along the river corridor, when new populations successfully established downstream from the places of their original introduction.

It is commonly considered that populations of native plant species should be used in revegetation (Smetanin 2000; Chibrik 2002; Timofeeva et al. 2016). However, the list of species tried and recommended for this purpose in Murmansk Region (Kryuchkov 1985; Druzhinina and Mialo 1990; Timofeeva et al. 2016) contains plants (*Bromusinermis*, *Loliumpratense*, *Phleumpratense*, *Alopecuruspratensis*) which are not native to Murmansk Region, and therefore their introduction contributes to a further pollution of this territory by alien plants. Moreover, these seeds are produced commercially by agricultural enterprises (Zolotarev et al. 2017) far away from the area of introduction. Use of commercial seeds collected in remote territories brings seed contaminants which may appear either as easily recognisable exotic aliens or as cryptic invaders masquerading as their native relatives. The latter case is exemplified here by the *E.acris* group, which provides an alarming example of new invasions that can be started even when the technical standards in revegetation have been duly followed, and then completely neglected because of the lack of knowledge in this taxonomically difficult group. In order to prevent such situations, we suggest that protection from invasive non-native plants should be more efficient at the stage of seed import, and monitoring and management of invasive non-native plants should be established in places of revegetation works.

### ﻿Putative hybridisation

So far, there is no direct confirmation of interspecific hybridisation in the *Erigeronacris* group which is based on genetic studies or experimental crosses. However, the existence of putative hybrids has been noted by a number of researchers who attempted to define taxa more precisely in this group.

Botschantzev (1959), who accepted only two taxa of the *E.acris* group in the Russian North, the hairy *E.acris* and the glabrous *E.politus* (which he named *E.elongatus*), speculated that individuals with presumably intermediate morphology originated from hybridisation between these two species. He also suggested that *E.brachycephalus*, *E.droebachiensis*, *E.elongatiformis* and *E.uralensis* may have the same hybrid origin. However, the specimens at LE collected from Murmansk Region, which Botschantzev identified as hybrids, mostly belong to *E.politus*, except for two specimens of *E.rigidus*, one specimen of E.×pilosiusculus and one specimen of *E.brachycephalus* that do not show any regular pattern.

Tzvelev (1990, 1994) agreed with Botschantzev’s hypothesis of intermediate origin of these taxa, except for *E.droebachiensis*. He lumped these taxa together as a single species, which he named *E.uralensis* by priority, and expanded it by adding *E.decoloratus* as a further synonym. Like Botschantzev (1959), Tzvelev (1990) also noticed presumably hybridogenous individuals between his accepted taxa.

As *E.rigidus* possesses a seemingly intermediate morphology between *E.acris* and *E.politus* and its main distribution area has an altitudinal character and lies between the areas of these two species, we speculate that this species originated from ancient interspecific hybridisation. This idea may also explain the more recent hybridisation between *E.acris* and *E.rigidus* and between *E.politus* and *E.rigidus* in the places of their current co-occurrence. This hybridisation is also inferred from intermediate morphology of co-occurring individuals, which are regularly collected together and placed on the same herbarium sheets by collectors.

So far, we detected several localities with hybridisation between *E.politus* and *E.rigidus* (Kandalaksha Gulf, Turii Mys, Varzuga, the mouth and lower course of Ponoi River) and two local areas of hybridisation between *E.acris* and *E.rigidus* (Tetrino Village, Apatity Town). We expect that the hybridisation may be even more widespread and complicated but it cannot be studied in full on the basis of the morphology of historical herbarium specimens.

## ﻿Conclusions

Our treatment is a pilot study on the *Erigeronacris* group in Eastern Europe that covers a single first-level administrative subdivision of Russia, which was selected due to its extreme northern position that allows easier detection of introduced plants. The territory of Murmansk Region is a fair representative of the Lapland flora; it makes possible to decipher the taxonomic composition in the Fennoscandian North with this territorial example. Murmansk Region is a meeting point for the western (Atlantic) and eastern (Siberian) flora (e.g. Kremenetski et al. 1999) and southern (Hemiboreal) flora (e.g. Korsakova et al. 2021), with a rich and diverse alien component which has arrived mostly through southern pathways (Kozhin et al. 2020b).

The present contribution puts forward a morphology-based hypothesis about the taxonomic structure, distribution and history of the *E.acris* group in the Russian North. We provide the following major conclusions:

1. The taxonomic diversity can be classified into two main groups, which are characterised by their synflorescence structure: plants with corymbose synflorescences (corresponding to *E.* ser. *Trimorpha*) and plants with paniculate synflorescences (corresponding to *E.* ser. *Macrophylli*).
2. Plants sharing the same type of synflorescence but characterised by a various density of pubescence (ranging from nearly glabrous to moderately pilose and, ultimately, to densely hairy) are closely related and may be connected by hybridisation, and for this reason should be classified in the same group. The taxonomic separation of subglabrous taxa (corresponding to *E.* ser. *Politi*) is not supported.
3. The regional taxonomic diversity has been dramatically underestimated. This was caused by taxonomic confusions and the lack of taxonomic expertise, as well as by numerous recent introductions. Some taxa previously treated as synonyms should be restored (*E.brachycephalus*, *E.rigidus*). On the other hand, a number of previous new records were based on misidentifications (*E.decoloratus* should be excluded as reported in error, earlier records of *E.acris*, *E.droebachiensis* and *E.uralensis* were erroneous).
4. *Erigeronacris* of the current treatments in Eastern Europe and Fennoscandia contains two distinct taxa: *E.acris* s.str., which is more hairy and green, common in the southern part of Fennoscandia, and *E.rigidus*, which is less hairy and purplish, reaching the northern part of Fennoscandia. This morphological and biogeographical distinction is very clear and unambiguous.
5. The distribution of the only native species in the area, *E.politus*, is limited to the mountainous or hilly areas, or to the territories with deeper river valleys. The distribution of old introduced taxa (archaeophytes and old neophytes) is restricted to the area which has been traditionally inhabited or used by Russian settlers. The distribution of new introduced taxa (recent neophytes) is limited to industrial areas.
6. The present-day picture of a high taxonomic diversity and extensive distribution of alien taxa was caused by a combination of long-distance and local dispersal events. The first major cause of introduction and further dispersal of alien taxa were Russian seashore settlers, who have inhabited the territory for several centuries and carried the diaspores along their fishing and trade routes. Among the taxa introduced and dispersed in this way, the naturalisation of *E.rigidus* is the earliest, following by *E.brachycephalus* and *E.acris*. The introduction of *E.acris*, *E.brachycephalus*, *E.droebachiensis* and *E.uralensis* occurred from remote territories with revegetation of industrial areas (dams, tailings and slag-dumps).
7. Considering the means of introduction, we assume that the main historical pathway was dispersal of diaspores by transport (by vehicles and then by feet) rather than with contaminated items. This conclusion agrees with the transportation and dispersal of the diaspores by fishermen who were not known to carry any significant cargo during their activities. The observed historical introduction was not connected with agriculture or hay-making either. The main modern pathway was seed contamination, coupled with long-distance dispersal by transport.
8. When native and introduced taxa of *E.* ser. *Trimorpha* come into contact, individuals with intermediate morphology may be observed in the same localities. These intermediates presumably originate from hybridisation, which may cause gene pollution in native (*E.politus*) and introduced (*E.rigidus* and *E.acris*) taxa, posing another threat to the native biodiversity (Bleeker et al. 2007). Besides, alien species may outcompete their native close relatives due to shared ecological niches (Pouteau et al. 2023).
9. This morphology-based taxonomic hypothesis provides a background to future phylogenetic studies on these groups, which should also take into account the variability (both infraspecific variability and introgressive hybridisation) and complicated history of human-mediated dispersal.

In comparison to the previous treatments, our taxonomic concept most closely corresponds to the ideas of Šida (1998). Like Mela (1877, 1884, 1895, 1899), we accept a single major subdivision of the *E.acris* group but use the synflorescence structure as the main character (Šida 1998). Our taxonomic revision closely corresponds to the latest Finnish synopsis (Kurtto and Väre 1998) in the species circumscriptions (with the additional separation of *E.rigidus*) but differs in the nomenclature; it can be considered an expansion of the Finnish treatment northwards. Our revision is also a development of the treatment for Eastern Europe (Tzvelev 1994), which differs in a coarser taxonomic resolution. The taxonomic revision of Olander and Tyler (2017), which was based on a statistical analysis of morphological characters, differs from any previous treatment and is largely incongruent with our conclusions due to its lumping approach.

Although taxonomic treatments produced for smaller territories (like Murmansk Region) seem to be limited in their scope, they can achieve very detailed, reliable and therefore useful results when placed in a broader context. For example, our revision of *Erigeronannuus* L. s.l. in Eastern Fennoscandia (Sennikov and Kurtto 2019) covered a small portion of its global distribution area but took the global studies into account, which facilitated further revisions in Europe (Otto and Ferloove 2019; Gudžinskas and Taura 2020; Sennikov and Galasso 2021) and Asia (Sennikov et al. 2020; Sennikov and Lazkov 2021). We hope that the results of our present study pave a path to further detailed revisions of the *E.acris* group in Fennoscandia and Eastern Europe.

As the next step, we welcome further cooperation to confirm the taxonomic structure proposed in this work by phylogenetic methods. As long as reliable phylogenes are not available, the validity of our conclusions is confirmed by the match between plant morphology and historical processes uncovered in our work.

## Supplementary Material

XML Treatment for Erigeron politus

XML Treatment for Erigeron × pilosiusculus

XML Treatment for Erigeron rigidus

XML Treatment for Erigeron × intercalaris

XML Treatment for Erigeron acris

XML Treatment for Erigeron droebachiensis

XML Treatment for Erigeron uralensis

XML Treatment for Erigeron brachycephalus

## ﻿Additional information

### Author ORCIDs

Alexander N. Sennikov https://orcid.org/0000-0001-6664-7657

Mikhail N. Kozhin https://orcid.org/0000-0002-0153-0287

### Data availability

The dataset of distributional records collected for the present work was published through the Global Biodiversity Information Facility (available online: https://doi.org/10.15468/een8vj).
